# Theory and rationale of interpretable all-in-one pattern discovery and disentanglement system

**DOI:** 10.1038/s41746-023-00816-9

**Published:** 2023-05-22

**Authors:** Andrew K. C. Wong, Pei-Yuan Zhou, Annie E.-S. Lee

**Affiliations:** 1 Systems Design Engineering, University of Waterloo, Waterloo, ON Canada; 2 Computer Science Department, University of Toronto, Toronto, ON Canada

**Keywords:** Machine learning, Health care

## Abstract

In machine learning (ML), association patterns in the data, paths in decision trees, and weights between layers of the neural network are often entangled due to multiple underlying causes, thus masking the pattern-to-source relation, weakening prediction, and defying explanation. This paper presents a revolutionary ML paradigm: pattern discovery and disentanglement (PDD) that disentangles associations and provides an all-in-one knowledge system capable of (a) disentangling patterns to associate with distinct primary sources; (b) discovering rare/imbalanced groups, detecting anomalies and rectifying discrepancies to improve class association, pattern and entity clustering; and (c) organizing knowledge for statistically supported interpretability for causal exploration. Results from case studies have validated such capabilities. The explainable knowledge reveals pattern-source relations on entities, and underlying factors for causal inference, and clinical study and practice; thus, addressing the major concern of interpretability, trust, and reliability when applying ML to healthcare, which is a step towards closing the AI chasm.

## Introduction

Applications of machine learning (ML) in healthcare require accurate and reliable solutions to detect diseases at an earlier stage to recommend treatments based on an understanding of the supporting evidence. For high stake human-oriented applications such as healthcare, the judicial system, and human resource recruitment, ML models have to be transparent for interpreting their predictions and explaining decision boundaries^1–8^.

Why is it difficult for existing ML models to meet these needs? For decades, ML and data mining on relational datasets, represented as large tables with rows as entities, columns as attributes (features), and entries as attribute values (AVs), have been based on obtaining associative and discriminative information from the entity and relating them to the underlying ground truth or primary source. For example, decision trees extract rules from entities as decision paths, and Artificial Neural Networks extract weights on the layers. Association rule mining discovers simple patterns (i.e., itemset) associated with specific classes, but often produces too many patterns that cloud interpretability. Since feature value associations (patterns) caused by different underlying factors could be interwoven in an entity, the resultant associations are entangled^9^ and thus difficult to be directly related to separate interpretable sources.

In order to meet the critical scientific, medical, legal, and societal needs, the identified requirements for the ML models are: (a) transparency^10,11^ and knowledge organization^12^ to allow interpretability and traceability^13^; (b) improvement of classification results not relying on intensive training and feature engineering; (c) identifying and correcting label discrepancies^10,14^; (d) discovering rare and new classes and mitigating class imbalance problems^15,16^. If these needs are met, a clinician can visualize the causal relationship between an input and an output and understand the inference and evidence produced by an ML model, then trust is established. This requires the ML model to produce robust, stable, statistical verifiable and interpretable results with explicit visualizable functional as well as relational information^17^.

Today, the most powerful learning methods cannot explain succinctly why certain results are obtained. Holzinger^18^ reviews a collection of interpretability methods that rely on domain-specific knowledge. But such approach is disadvantageous when the collected data or ground truths is unreliable, such as the effects of racial bias^17^. Sometimes, the ground truths cannot be established due to either lack of quantitative modeling or generalizable medical research, such as in cancer detection and diagnosis^17,19^. Thus, expecting perfect ground truth and class labels in healthcare data is unrealistic^17,19^. ML models are also required to accept imbalanced data and prevailing errors, even those are deemed to happen. Due to the shortcomings in the field, we created PDD to resolve these problems.

PDD originated from the astounding finding in our bioinformatic research on amino acid interactions between binding proteins. We found from a large volume of laboratory data^20^ via X-ray crystallography, that the statistics of different types of physicochemical interactions between amino acids in two binding sequences shows confusing patterns. We then invented an association disentanglement algorithm via principal component analysis (PCA) to obtain the statistics of different distinct physicochemical interactions^21^. Later, we extended this approach to relational data in the early form of PDD^9,22^. Our early success and realization of the broad potential of PDD motivated us to embark on the journey to validate the ideas of PDD via pilot studies using well-studied datasets from ML communities to cover different problem domains and applications. Based on the early successful results and new insight of our pilot studies^9,23,24^, we embarked an in-depth study to obtain a thorough theoretical justification and experimental validation. We then identified the fundamental changes (a paradigm shift) of PDD and integrates its various capabilities into a collection of self-error-correcting class association and entity clustering algorithms. We hereby communicate our newly finished work to the ML and healthcare communities to convey the usefulness of PDD to further its advancement.

**Fig. 1:**
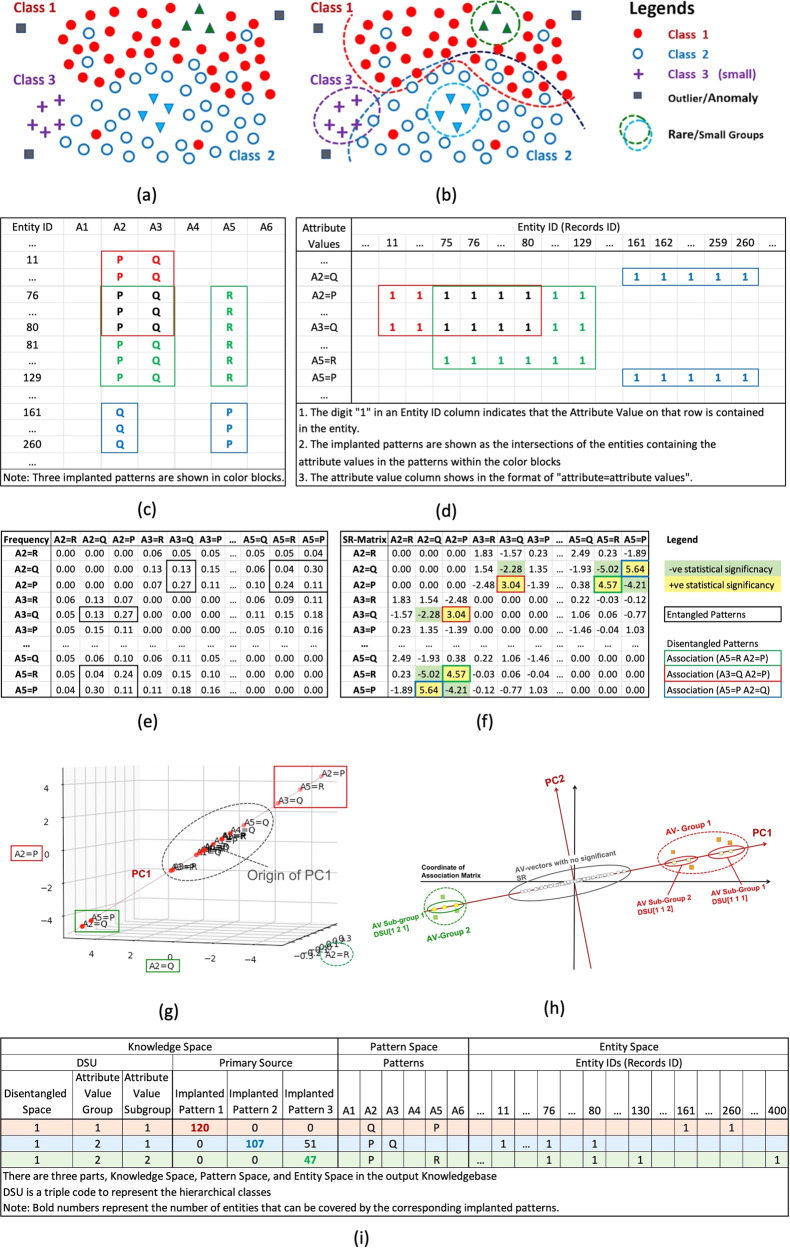
Conceptual flow diagram illustrating key elements in PDD. **a** Feature vectors mingle in the feature space due to partial overlaps of source-related AV associations in the entities. **b** Association disentanglement separates entities based on the disentangled patterns found in the disentangled association space (DS). Mislabels (blue dot amidst the red dots) contain patterns of the red dot. Class 1, 2, and 3 are imbalanced. The square dots are outliers. **c** We use the synthetic dataset with three implanted AV associations (marked in distinct color borders) to illustrate the use of Entity ID Address Table. **d** In the table. for each AV, we list on its row by the digit “1” the Entity IDs of the entities containing that AV. The green block (from EID75 to EID129) shows the intersection of the entities containing the AV-Group (A2 = P, A3 = Q, and A5 = R) with the frequency of occurrences = (129-75 + 1) = 55. **e** The Frequency Matrix with relative frequency of AV pairs show that the pattern (A2 = Q, A5 = P) and subpattern (A3 = Q, A5 = R) on row 2 are entangled. **f** The statistical residual matrix is obtained from the frequency matrix by converting its frequency into statistical residual (Table 1, Definition 1). Each row of the SR-Matrix corresponding to an AV is referred to as the AV vector of that AV with the SRs of its association with other column AVs as coordinates. **g** The first principal component (PC1) contains 2 AV-Groups (A2 = P, A3 = Q, A5 = R) and (A2 = Q, A5 = R) on the extremal ends of the PC, indicating strong in-group AV-association. AVs close to the PC origin have weak AV associations with other AVs. **h** Two AV-Groups are obtained on the PC1. AV-Group 2 contains two subgroups. Each sub-group is a unit of disentangled space with triple code in the square brackets, e.g., DSU[1 2 1] represents the first pattern sub-group, within the second pattern group in the first disentangled space. **i** The Summarized knowledge base consists of the Knowledge Space encompassing the DSU with discovered disentangled pattern(s) occurring on the entities in the Entity Space as displayed in the Pattern Space.

Figure 1 illustrates the problem. Traditional ML models attempt to find the feature- value-associations in the entities associated with distinct sources. However, when they are overlapped in entities, those entities in the feature space may be blended near the decision boundary between different groups (like those between Class 1 and Class 3 on the lower right of Fig. 1a). We say that the patterns from different sources, such as source and receiver in classical Shannon theory (or signal processing) are entangled in the entity. It takes extensive effort to separate the entangled association, and/or discriminative information from the entities. Therefore, we developed pattern discovery and disentanglement (PDD) to solve these problems. To obtain associations relating to sources (Table 1), we use principal component analysis (PCA) and statistical associations. Traditional application of PCA does not seek interpretation of the PC or the source. But when we find statistically connected AV groups (patterns) captured by the PC occurring on entities associated with specific characterises, functionality or classes (like those found in all of our cases studies), we can consider them as the primary sources associated with the disentangled patterns that the entities possess. In another word, we identify the source via the entities. Such relations can be validated.

**Table 1:** Terminologies and definitions.

Terminology	Definition	Practical application examples
Attribute values (AV)	Categorical value of an attribute (i.e., nominal, or quantized interval values of numeral data).	Traits of patients.
Attribute value association	An AV-association is referred to as a group of AVs co-occurring in an entity.	Co-occurring traits of heart disease.
Pattern	Patterns are statistically significant AV associations of several AVs.	Statistically significant traits of heart disease in a patient.
Entanglement	AV associations or patterns coming from more than one class co-occurring in the same entity, which is not readily separable.	Symptomatic patterns of different diseases are found in the same patient.
Source	An underlying physical reality or contribution factor (functional/circumstantial) presumed to cause a specific pattern to occur in entities.	Etiological cause of a disease; or a taxonomic class.
Address table	A table listing, for each distinct AV in the dataset, the IDs of the entities embodying that AV.	Fig. 1d
Statistical residual (SR)	The statistical residual is a measure of the statistical significance of the AV association. (Definition 1 in the Methodology)	The numeral 3.04 in Fig. 1f is the SR of the pair (AV (A2 = P) and AV (A3 = Q)), applying to a group of AVs.
SR-matrix	A matrix of statistical residual (SR) for each pair of AVs for evaluating the statistical strength of the association between AVs.	Fig. 1f is a part of the SR-Matrix containing the SR values for each attribute value association.
Attribute value vector (AV-vector)	A row in the SR-Matrix is referred to as the AV-vector whose coordinates are the SRs of that AV associated with other AVs corresponding to the column vectors.	Fig. 1g, h illustrate the vectors and their projections on the PC in the Reconstructed SR-matrix.
Principal component analysis (PCA) in this paper	A process of a linear transformation to decompose the SR-matrix (a set of AV vectors) into principal components (PCs). Each PC captures the AV associations which is functional independent to those captured by another PC.	Red axes in Fig. 2h.
Reconstructed SR-matrix	A reconstructed SR-Matrix containing the projection of AV-vectors on a principal component	Fig. 1f.
Statistically connected	Two AVs associated with statistically significant SR are statistically connected.	All AVs in an AV-subgroup.
Disentangled space (DS)	Disentangled space is a reconstructed SR-matrix containing statistically significant AV-associations	DSU[1 - -] in Fig. 4a is the DS from the first PC.
AV-Group	A group of AVs with statistically significant AV-associations.	DSU [3 2 1] stands for the 1st AV-Subgroup in the 2nd AV-Group discovered from the 3rd disentangled space (DS).
AV-Subgroup	A set of AV-subgroups clustered from an AV-Group based on the similarity between subgroups that is evaluated by the overlapping percentage of entities covered by the subgroups.	All AV-associations in DSU[1 1 -] in Fig. 4.
Disentangled space unit (DS-unit)	A DSU is a unit represented by a triple-code: [#DS, #AVG, #AVSG]. DS is the disentangled space; AVG is the attribute values group and AVSG is the attribute values sub-group.	DSU[3 2 2] with the 3rd AV-SG, 2nd AV-G of DS 2 in Fig. 1c is an DSU.
Entity cluster	A group of entities that are covered by the corresponding AV subgroup.	Figs. 3e, 4b.
Knowledge base (KB)	A knowledge representation consisting of: (1) a knowledge space: (with DSUs and number of patterns occurring in entities associated with their primary sources/classes); (2) a pattern space: (with patterns in each DSU), and (3) an entity space: (with EIDs and the patterns indicator and class status (if class label is given to each entity)	Summarized knowledge base contains union patterns obtained from all patterns discovered in each DSU. (e.g., Fig. 1i)

The objective and capability of PDD are as follows. PDD can: (a) efficiently discover compact sets of patterns associated with their sources through the entities; (b) discover statistically significant associations without the need for manual feature engineering, (c) combine supervised and unsupervised methods to exploit the ground truth while unbiased to overfitting and label discrepancies in the data to obtain auto-error-corrected class associations and entity clusters, (d) discover rare and new patterns, even from imbalanced data, and (e) produce a knowledgebase interlinking patterns, entities and sources for visualization, interpretation and further iterative exploration. This paper presents the theory and rationale of PDD, its major paradigm shifts from existing ML models, and its interpretable experimental results to validate its unique capabilities. Hence, to demonstrate the underlying workings of PDD, we give a brief description of an overview in algorithmic steps (the what), the theory (the how) and the rationale (the why) as below to enable the interpretation and understanding of the results.

**Fig. 2:**
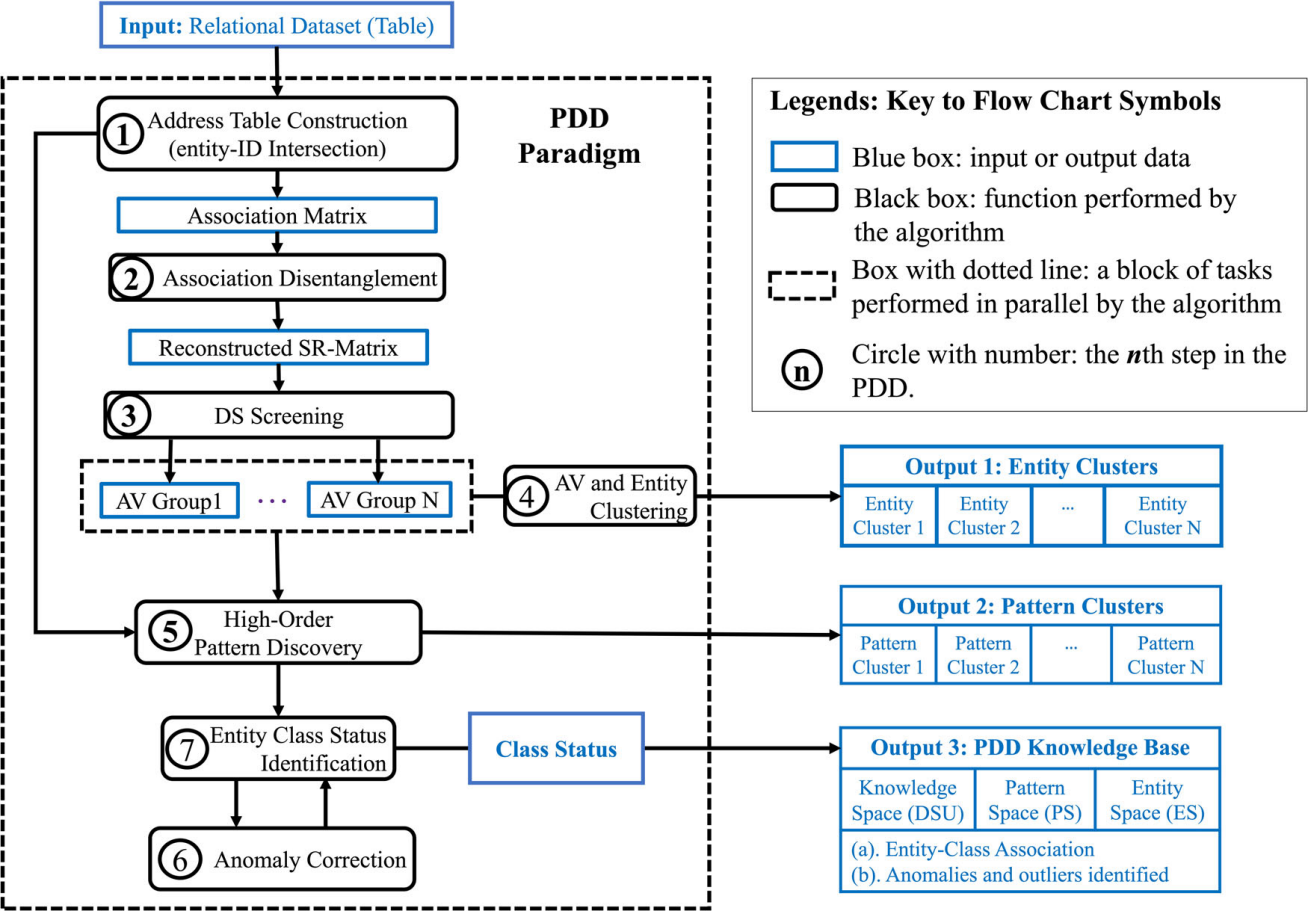
Overview of the PDD.

Table 1 provides the terminologies with definitions. Figure 1c, i illustrate key concepts of PDD with a synthetic dataset. Figure 2 shows the flow of the key processes in an all-in-one PDD system. Figure 1a illustrates that if AV association patterns from different sources or classes are overlapping (entangling) in some entities, then their feature vectors of different classes blended in the feature space are difficult to separate. However, if AV associations (disentangled patterns) associated with distinct classes found in disentangled spaces by PDD are mapped back to the entities, then their class status is ascertained as marked in Fig. 1b. They are separated.

As an illustrative example, we use an input dataset (Fig. 1c) with 6 attributes and 400 entities with randomly distributed AVs and 3 implanted associations. First, we change the combinatorial search process of AVs and AV groups directly from the dataset for pattern discovery to a simple table lookup and a sorting process. We create an AV Entity ID Address Table (Address Table) that lists, for each AV, the Entity IDs (EIDs) of all the entities containing that AV (Fig. 1d). From the Table, we obtain the co-occurrence frequency of two associated AVs from the set-intersection of their entity lists.

In the step 1, we use the standardized residual (SR, Definition 1 in Methods) to construct the SR-Matrix (Fig. 1f) by calculating the SR for each pair of AVs (Definition 2 in Methods). Each row of the matrix corresponding to an AV is considered as an AV-vector whose coordinates are the SRs of that AV associated with other AVs corresponding to the columns.

In the step 2, for association disentanglement, we apply PCA to the SR-Matrix to obtain a projection of its AV-vectors onto its principal components (PCs). For example, A2 = P, A3 = Q, and A5 = R (Fig. 1g) at a distance from the PC origin indicate their strong AV-association among themselves. Then, replacing the AV-vectors by their projections on each PC in the original SR-matrix, we obtain a set of new SR-Matrices each of which is referred to as the Reconstructed SR-Matrix for the PC reflecting the AV associations captured in the PC.

In the step 3, after obtaining the reconstructed SR-matrices, to guarantee the discovered associations are statistically significant, we filter those containing statistically significant SRs (Table 1) and refer them together with their corresponding PC as disentangled spaces (DS). We then group AVs into AV-Groups in each DS if each AV in the group is statistically associated with at least one other AV in the group.

From the theoretical standpoint, if an AV-Group is a consequence of a source and not a random happening, it should satisfy three conditions: (1) functional independent of other AV-association(s), (only on one PC); (2) statistically significant, and (3) separated from associations in other AV-Groups. To meet these conditions, we (1) apply PCA on the SR-Matrix to disentangle associations as functional independent AV associations; (2) use DS screening to guarantee that the discovered associations are statistically significant, and (3) ensure that no AV in an AV group within an entity is statistically connected to AVs of other AV groups.

We now explain why PDD association disentanglement works. Traditionally, PCA is used to transform data into a representation in which the principal dimensions are orthogonal to one another. Each dimension can be interpreted as representing a functionally independent underlying driver of the observed data. In this sense, decomposing the data into their principal component is a step toward disentanglement. The efficacy of this intuition is validated by the results produced by PDD. PDD applies PCA to obtain AV associations associated with the underlying source in an unsupervised manner unlike linear discriminant analysis (LDA), which is a supervised method applying PCA for discriminating entities between classes.

In the step 4, to obtain AV-subgroups separated from other subgroups, we apply a hierarchical clustering algorithm using the overlapping percentage of their covered entities as a similarity measure (see the Subgroup Clustering Algorithm). When an AV-Subgroup satisfies the three conditions, we conjecture that it is the consequence of certain primary sources. Then each AV-subgroup is referred to as disentangled space unit (DSU) with a triple code [#DS #AVG #AVSG] where “#” signifies the ordinal ranking of the item. The entities covered by the AV-subgroup can form an entity-subcluster to output it as an entity cluster.

In the step 5, to conduct a deeper analysis of the results, PDD obtains patterns from each DS containing statistically significant AV associations with and without using classes to fully exploit the ground truth while screening out biases. To discover statistically significant patterns, we take subgroups of AVs within AV-subgroup as pattern candidates to be confirmed as patterns via a pattern hypothesis test (Definition 10 in Methods). In pattern discovery, PDD obtains the frequency of occurrences of AV groups discovered in each DSU from the set-intersection computational process from the EID Table, not searching for candidates from the dataset. Patterns discovered in each AV-subgroup then naturally form a pattern cluster.

In steps 6 and 7, to achieve auto-error-correcting classification and entity clustering results, we use Step 7, with Step 6 in between to obtain class status for each entity with or without class label given. We assign four categories for the class status: (i) Correct (Cor) (entity contains the number of patterns of its given class greater than those of other classes); (ii) Class Label Re-Adjust (CRA) (entity contains a pattern of another class other than its labeled class); (iii) Undecided (Und) (entity contains an equal number of patterns of two classes) (iv) Incorrect (Inc) (entity contains patterns associated with the given class less than that of another class) (v) Outlier (OL) (no patterns discovered). After confirmation and adjustment, PDD marks the Entity-ID with the color code/icons of the adjusted class and outputs its class association results.

**Fig. 3:**
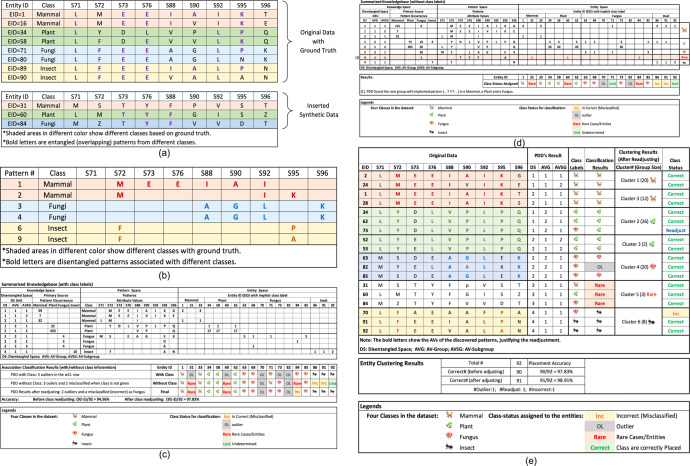
The Output of PDD for Cytochrome C APC data. **a** It is part of the input data showing the entanglement of patterns associated with different classes/sources within entities. A synthetic pattern S73 = T, S6 = Y, and S88 = F are implanted on a Mammal, a Fungus, and an Insect. **b** It shows PDD separating the statistically correlated patterns occurring on entities in the original data into *disentangled patterns* pertaining to distinct sources/classes. **c** It shows the summarized Knowledgebase with class label given. Note that the union of the disentangled patterns discovered in each DSU occurred only on entities related to one class/source—indicating perfect disentanglement. **d** In the Knowledgebase from no-class-label dataset, the knowledge space shows that the union pattern in DSU[1 1 1] is shared by Mammal and Fungus, revealing their common functionality. However, when PDD was applied to these entities, patterns associated with Mammal are separated ((**c**) in Supplementary). In (**c**), PDD discovered 5 outliers. In (**d**), unaffected by class, we found 3 entities formed a rare group with the implanted pattern [T Y F] in DSU[3 1 1]. EID84, found also as Fungus (as labeled), we assigned that as its final status by Rule 1. PDD attained a class association accuracy of (95-3)/95 = 97.83% after result integration. **e** In the abridged entity cluster, each row is an entity. The PDD Results columns show the DSU of the discovered pattern(s), followed by its class label and the final classification results (**c**), and the number and the class status of all the entities placed in each cluster after the readjustment. The last column displays the cluster placement status - correct (cor), readjust (after Cra), and Inc when the entity is placed into a wrong cluster. After class correcting (Cra), only EID70 and EID73 were found misplaced. Since EID73 was originally labeled as Fungus but found to contain patterns associated with Plant instead, it was considered as a Plant. Hence, with only one error, PDD obtained a placement accuracy of 98.91%.

The output of PDD obtained from a dataset with and without class label included is a well-organized interpretable knowledge base, interlinking the knowledge space, pattern space and entity space (Fig. 1i). *Knowledge Space* consists of DSUs and the distribution of the number of their AV-Groups occurring on entities associated with different primary sources, to reveal how good the disentanglement is, like (DSU[1 1 1]). *Pattern Space* displays the patterns in each DSU. *Entity Space* lists all entities with distinct Entity-ID, and the pattern(s) each possesses by the “digit” on its ID column and DSU row (Fig. 3c). It also displays the class status assigned to each entity after error correction.

After the most fundamental disentangled-associations are attained, PDD uses them to solve various ML problems in a comprehensive all-in-one process. It accomplishes auto-correcting class association and entity clustering even with imbalanced, new, and rare groups of low-quality and low-volume data with high accuracy. PDD produces a knowledge base that summarizes yet retains all information interlinking patterns, entities, and primary sources for interpretation, causal exploration, and knowledge extension. It is computationally efficient with low time complexity executable in parallel mode. As shown in the results, it is efficacious when applied to various types of biomedical and clinical problems. In summary, it paves the path to close the AI chasm.

## Result

### Background and objective of the case studies

In order to highlight the biological and medical applicability of PDD, we used *cytochrome c*^25^ (structured tabular output from the Aligned Pattern Cluster (APC) process) and Heart Disease Dataset^26^. We used these experimental results to show and explain PDD’s efficacy and capabilities. The *cytochrome c*^25^ data was selected because it provides explainable and verifiable scientific evidence. We chose heart disease dataset^26^ because it is the benchmark dataset that is easy to explain and to validate the key capabilities of PDD in comparison with other ML models. The other datasets, APC of Class A Scavenger Receptor^25^, Wisconsin Breast Cancer^27^ and Thoracic Surgical Risk^28^ in the Supplementary were chosen based on the same consideration. Three of the datasets we used are benchmark datasets, and two with strong scientific justification so that the results are easily understood with comparison with other existing ML methods (Fig. 5) and our published work in proteomics^23,25^. Table 2 and III show the capability and results comparison of PDD with other models. In Fig. 5, we took from the literature the best results of the well-studied heart disease data in the ML community for comparison.

**Table 2:** Capability comparison.

No.	Machine learning tasks	PDD	Unsupervised Learning Algorithm (e.g., K-means)	Supervised Learning Algorithm (e.g., SVM, ANN…)	Association Mining/Classification (DT, Apriori, Association Classification…)
1	Classification (supervised learning)	YES	NO	YES	YES
2	Imbalanced classification (supervised learning)	YES	NO	Partial YES (need sampling/extra processing for dataset)	Partial YES (need sampling/extra processing for dataset)
3	Clustering patterns (unsupervised learning)	YES	NO	NO	Partial YES (need extra pattern pruning/pattern clustering)
4	Clustering entities (unsupervised learning)	YES	YES	NO	NO
5	Interpreting associations with rules or patterns	YES	NO	NO	YES
6	Discovery rare patterns	YES	NO	NO	Partial YES (need customized threshold)
7	Detecting rare cases, outliers, anomalies	YES	NO	NO	NO
8	Detecting mislabels & rectifying classification	YES	NO	NO	NO
9	Transparency & explainability of throughput/output	YES	NO	NO	NO
10	Knowledge discovery: relating disentangled patterns to known or unknown sources	YES	NO	NO	NO
11	Explainable & extendable knowledgebase	YES	NO	NO	NO
12	Supporting expert (tracking, monitoring, exporting)	YES	NO	NO	NO
13	Feature extraction/selection	Automatic significant AV selection	Need extra process for feature engineering.	Need extra process for feature engineering.	Need extra process for feature engineering.

Item 13 PDD automatically conduct feature engineering at the attribute value level via pattern disentanglement and obtaining subgroups of attribute values from disentangled space.

### APC of *Cytochrome C* and class A scavenger receptor family

The APC data was obtained from conserved functional regions of *cytochrome c* with imbalanced class size (Mammals 32, Plants 28, Fungi 22, and Insects 7)^25^. Figure 3a shows entities with entangled patterns. For example, EID1 of Mammal contains a pattern [E E I A] overlapping with AVs of Insects (EID89); AVs and the sub-pattern [- **F** E E **A G L A** P -] in EID80 *Fungus* overlaps with [- **F** E E - - **L A** -] in EDI90 *Insect*. Besides, we synthetically added 3 entities by implanting a common pattern [S73 = T, S76 = Y, S88 = F] into a *Mammal*, a *Plant*, and a *Fungus* while keeping their class label. Our objective is to validate PDD capabilities in finding AV-associations related to the source and to exemplify other PDD capabilities as well. Figure 3b shows that the disentangled patterns pertain to a single class (a primary source).

We used wCL and nCL to represent the dataset with class labels included or not included or the results obtained from them respectively. In wCL (Fig. 3c), PDD attains perfect disentanglement (i.e., every DSU contains union patterns pertaining to a single class), whereas in nCL, (Fig. 3d) the classification accuracy drops. However, PDD discovered the pattern […T R F…] in E31, E60, and E84; a fungus pattern in E73; and a pattern in DSU [4 1 1] for a small insect subgroup (E86, E91, and E92), which were found only as independent outliers in wCL (Fig. 3c). Note that EID84 was found as Fungus in wCL but as a rare group in nCL (Fig. 3d). By Rule 1, it was assigned as a Fungus. This indicates that PDD can unveil deeper knowledge not biased by class labels through unsupervised learning.

Figure 3e displays the abridged entity clusters results. In the original data columns, we displayed the EID of the representative entities with their AVs in each cluster. In the PDD Results columns, for each entity, we displayed the DSU where its discovered pattern(s) (in bold font in the Original Data columns) was (were) found. In the next two columns, we showed its original class label and the final class status assigned to the entity (from the row Final in Fig. 3c). In the clustering results column, we displayed the final class-status and the number of the entities being placed into each cluster (e.g., Cluster 1 contains 20 Mammals). We then displayed the cluster placement status of each entity in the last column—an entity placed into a correct cluster (based on the class label of its majority members) as “correct”, misplaced as Inc, and those corrected after Cra as “Readjust”. After Cra, only EID70 and EID73 were found misplaced. EID73 originally labeled as Fungus was found to contain patterns associated with Plant, it was considered as a Plant. Hence, with only one error, PDD obtained a placement accuracy of 98.91%. Unaffected by class, PDD obtained the rare group with pattern [T Y F] as a distinct cluster (Cluster 5).

For a more sophisticated APC of a diverse family, Class A Scavenger Receptors (SEC R-A), with 5 subclasses and functional domains widely spread, PDD attained class association and clustering accuracy of 97.89% and 95.70%, respectively. It located patterns in various functional domains^23^. The synchronized knowledge base (Fig. 3d) and entity cluster results (Fig. 3e) validate PDD efficacious capabilities.

### Heart disease dataset

Next, we used a well-studied healthcare benchmark dataset on heart disease^26^ from UCI repository^29^ containing 270 clinical records with 13 mixed-mode attributes in two classes (Absence (Abs) or Presence (Prs) of heart disease) to expound PDD’s capabilities in solving a realistic interpretable clinical problem.

**Fig. 4:**
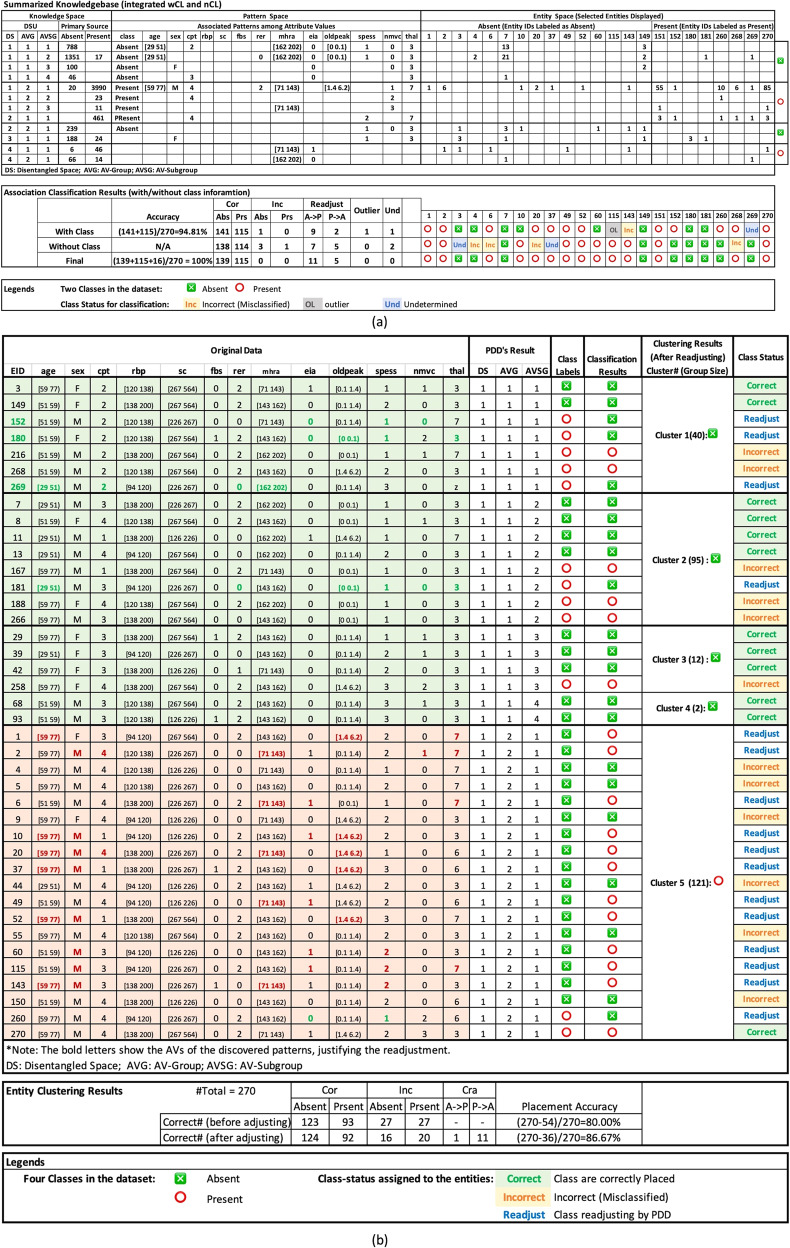
The output of PDD for Heart Disease Data. **a** In the DSU[1 1 1] of the Knowledge Space, 788 patterns within the union pattern in the Pattern Space were found occurring only on entities associated with Absence, indicating perfect disentanglement. A large variety of pattern orders was observed. In the Entity Space, the Entity IDs were sorted according to their implicit class—E1 to E150 are Absence, and E151 to E270 are Present. The class-status result was displayed on the “Final” row after correction. Note that EID7 possesses 13 lower-order patterns of the union pattern in DSU [1 1 1]. EID49, labeled as Absence, possesses no pattern of Absence but one of Presence. Hence it was adjusted as Presence after class readjusting. EID20 (of Absence) was found as Presence from wCL though also found as Inc from nCL, it was assigned as Presence (Rule 1). EID115 was an outlier in wCL as it possessed no significant pattern but was found as a Presence from nCL. Hence it was adjusted as Presence after class readjusting (Rule 2). EID268 was found as a Presence in wCL but as an Inc in nCL. It was assigned as Presence (Rule 1). EID270 was found to contain Presence patterns in both wCL and nCL (from implicit class), it was assigned as Presence (Rule 1). Overall, based on statistical patterns, PDD obtained an accuracy of 94.81% before class readjusting and 100% after according to the statistical rules (see discussion in the Supplementary). **b** In abridged entity clustering results, the PDD Results columns display the DSU with pattern(s) of each entity, the original class label and then the final class-status after class-correction. Attribute-values with green or red font indicates that they comply with the patterns of the correct class after adjustment. As the font color shows, EID152 and EID180 pertain to patterns of Absence although being labeled as Presence. In EID1 and EID 2 were found pertining to Presence though being labeled as Presence. The last column displays the cluster placement status. The results show that the accuracy before class readjusting = (270-54)/270 = 80.00% and = (270-36)/270 = 86.67% after.

Figure 4a displays the summarized knowledge base obtained from data with or without class given. In the Knowledge Space, we found in DSU[1 1 1] all the disentangled patterns, represented by the union pattern pertaining to only one class (Absence), indicating perfect disentanglement. EID1 to EID150 are Absence and EID151 to EID270 are Presence (Fig. 4a). Note that EID7 possessed 13 low-order patterns, all pertaining to Absence. In the Entity Space, the Entity-IDs were sorted to denote the implicit class of each entity. It showed patterns possessed by entities in the Entity-ID columns and displayed the integrated class status according to the Class-Association Rules below.

1. An entity is assigned as a Cor (Correct), an Inc (Incorrect) a Und (Undecided) if that is found from wCL in the consistency check. They are readjusted to class if found and confirmed from the results in either wCL or nCL (Rationale: PDD gives strongest weights to the ground truth unless label discrepancy is spotted).
2. An entity is an Outlier (OL) or an Undecided (Und) if found in both wCL and nCL but assumes the class status as found in either one of them.

By considering Inc and Und as incorrect, PDD achieved an accuracy of 94.81% before class readjusted and 100.00% after. As shown on the first row (With Class) in the Class Association Results (Fig. 4a), EID7 and EID270 are good examples of Cor. EID2 and EID52 were labeled as Absence but found containing only the Presence pattern. They were readjusted as Presence. As for the misclassified EID143 in wCL, it was discovered as a Presence via class readjustment in nCL and thus assigned as Presence by Rule 1.

Figure 4b shows the entity clustering results, using error-corrected class-status to obtain cluster placement accuracy. An entity is considered misplaced (incorrect) if placed into a cluster with the class of its majority members not complying with its implicit class. Figure 4b shows 54 entities misplaced before class readjustment and 36 after with 80.00% and 86.67% accuracy accordingly. Note that the clustering results are inferior to the classification results for it is an unsupervised method. But the error-corrected results in the Knowledge improve its accuracy. The inferior unsupervised learning results show that in the heart disease dataset, the assigned class label to the patients play a significant classification role.

### Other results

**Fig. 5:**
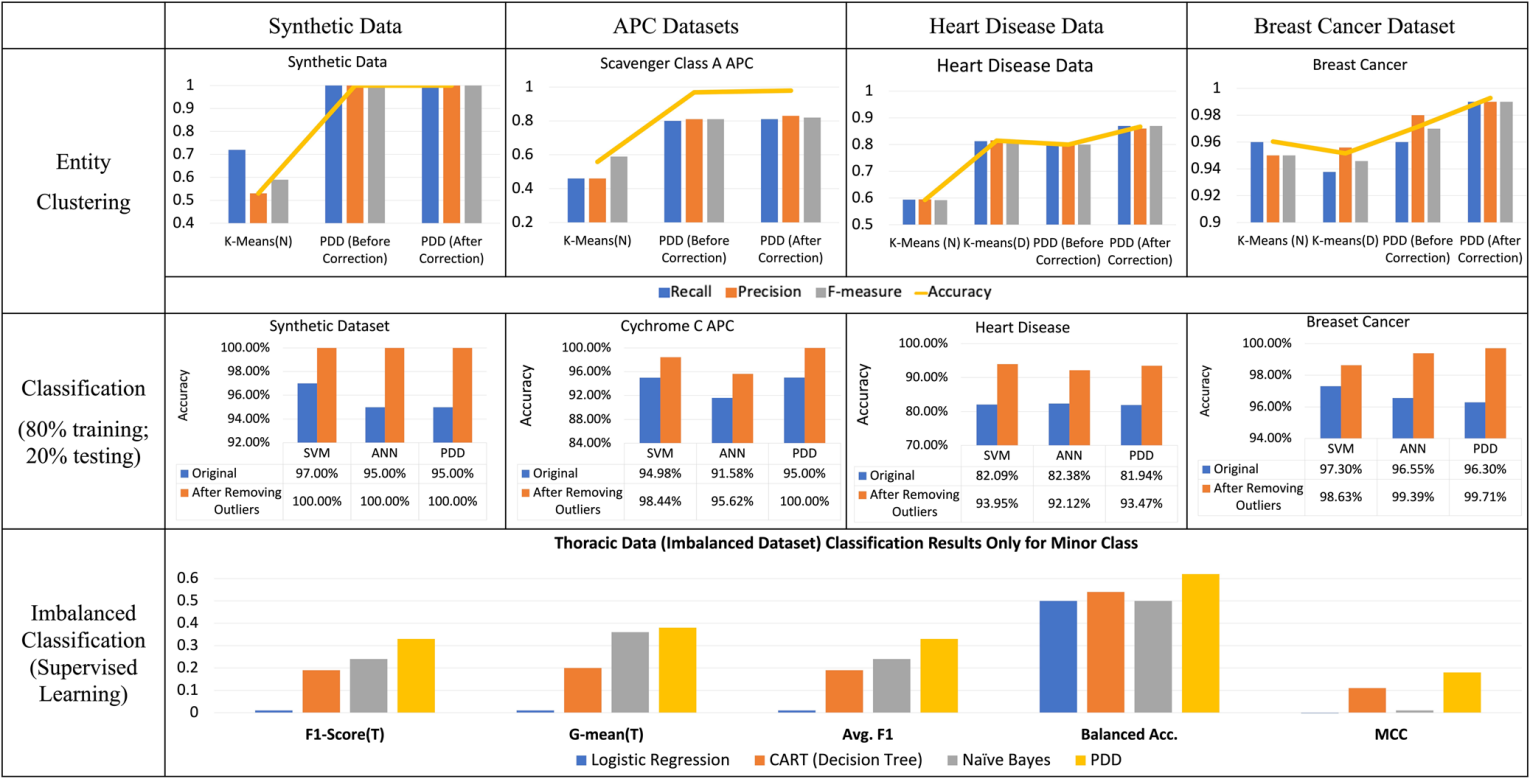
Results Comparison of PDD and other ML Models. In entity clustering, PDD outperformed K-Means (N) and K-Means (D) before and after error correction in recall (blue bar), precision (red bar), F-measure (gray bar), and accuracy (the orange line). In Class Association, note that the improved results in other ML models represented by the red bars were made possible when the outliers identified by PDD were removed. Without that, their accuracies were represented only by the blue bars, while those of PDD were represented by the red bars. The results show that in class association, PDD outperformed existing popular ML models significantly after class readjustment. Even before class readjustment, its class association results are comparable to those obtained by the existing supervised models. For the Classification of Imbalanced Classes, PDD (orange bars) outperformed other methods such as Logistic Regression, CART (Decision Tree), and Naive Bayes significantly.

In addition, all comparison results of various datasets from six case studies are summarized in Fig. 5. The results of three other datasets fully reported in the Supplementary are briefed here to show the broad applications of PDD on various types of real-world biomedical problems.

The first set is on the Wisconsin Breast Cancer data^27^. PDD obtained class association accuracy of 95.42% on wCL before correction and 99.86% after, see Supplementary Fig. 6; and 97.29% for entity clustering before correction and 99.28% after, see Supplementary Fig. 7. Column 1 displays the color code of the implicit class of the majority members of each cluster. Columns 3 and 4 display the implicit and the adjusted classes before and after class readjusting, respectively. The entity cluster placement accuracy before and after class status readjustment was obtained based on the given class and readjusted class (Column 4) respectively. It is marked in the last column as “Cor” if it is correctly placed, “Misplaced” if placed into a cluster with class not of its implicit class.

The Thoracic Surgical Risk dataset^28^ is an imbalance dataset when the Class Risk was assigned to a small fraction of the patients. Without correction, PDD obtained an association accuracy of 95.25%, biased toward the “no risk,” and an accuracy of 68.57% for “risk,” resulting in average and balanced accuracy of 91.27% and 82% respectively.

The third is a synthetic dataset to show the noise tolerance capability of PDD. Variable noise level was implanted in the data to provide a viable exemplification of noise tolerance of PDD. In both class association and entity-clustering, PDD outperformed its contemporaries after correction.

## Discussion

In the discussion, we summarize the unique capabilities of PDD (as itemized in curl brackets in Table 2) and other places, in comparison with other ML models; and, its noticeable improvement of classification and clustering results before and after its auto-error correction process (Table 3). They were all validated by the experimental results reported in the Main and the Supplementary. Figure 5 displays the quantitative comparison, showing that PDD, by and large, outperforms other ML models. We then discuss its impact in ML as well as in biomedical research and clinical practice with the unique capability of PDD as itemized below.

**Table 3:** Comparison of PDD performance in class association and entity clustering before and after auto-error-correction.

Accuracy summary	Before class readjusting	After class readjusting
(a) Classification (based on class association)		
Cytochrome c	94.56%	97.83%
Scavenger Class A APC	96.84%	97.89%
Breast Cancer	95.42%	99.86%
Heart Disease	94.81%	100%
(b) Entity Clustering		
Cytochrome c	97.83%	98.91%
Scavenger Class A APC	95.70%	95.70%
Breast Cancer	97.29%	99.28%
Heart Disease	80.00%	86.67%

Firstly, PDD uses PCA and SR to obtain a small set of DSs containing disentangled patterns on entities associated with known or unknown primary sources. This is unique. In all our experiments, the disentangled patterns obtained in DSs are associated with verifiable primary sources. Patterns interlinking entities and sources play a vital role in PDD.

Secondly, From the consistency of the discovered patterns and the explicit and/or implicit class label on individual entities, PDD identifies and corrects class discrepancies, and readjusts the class status of the entities for class association in a unique way as validated in the results (Figs. 3c, 4a, 5 and Table 3) {1, 2, 5–8}.

In addition, PDD uses its auto-error-correcting module to classify entities based on the disentangled pattern(s) in DSU {1}, not much affected by the entity group size or where the group is in the data. Hence, it classifies small and imbalanced classes with high accuracy as evidenced in the results in Case Study 1 to 5.

Moreover, in PDD, patterns in each AV-Subgroup naturally form pattern clusters without additional process^8^ {3}. On each AV-Group, a hierarchical clustering algorithm obtains entity clusters simultaneously {4}. Case study results show PDD producing much less pattern clusters and outperforming K-Means in entity clustering by far (Fig. 5).

Furthermore, PDD outputs an all-in-one knowledge base interlinks disentangled patterns with entities and their primary sources or classes to provide an interpretable platform for incorporating other causal and functional information, verifiable within and beyond the PDD framework {5, 9–12}. Such capability is validated in all the results reported in the Main and the Supplementary. It meets the demand of interpretability in clinical ML and offers a verifiable realistic platform for better understanding and assurance of the ML process and results at various levels, extendable for further exploration (See Figs. 3, 4, and Supplementary Fig. 2).

Finally, PDD changes the algorithmic approach in ML in a unique way. From disentangled AV-associations, PDD (i) uses a very small set of succinct and targeted AV-Groups from a small set of DS containing significant AV-associations; (ii) employs simple look-up and a simple set-intersection computation process via EID Address Table (Step 1) on AV-Subgroups for pattern discovery (Step 5)—a great complexity reduction in pattern discovery; and (iii) gives compact yet comprehensive knowledge representation in an all-in-one system—a great reduction of space complexity and improvement of knowledge management in the output.

PDD is a new paradigm for Interpretable ML. It renders a statistically robust, interpretable, and extendable knowledge platform, solving challenging problems confronting ML in an all-in-one system. In clinical applications, it assists experts’ diagnostic, prognostic, and therapeutic decisions with explainable and traceable evidence to gain confidence and trust in ML closing the Chasm of Human and AI.

## Methods

### Introduction

Before we present the notations, definitions, and the equations of PDD, we first give a high-level conceptual view of the methodology. The following items (labeled with the header **M**) represent the methodologies we developed in support of the PDD paradigm. We link them to the definitions, algorithmic steps, and experimental evidence. Specifically, we, via M1, use statistical residual (SR) (Definition 1), SR-Matrix and AV-Vector (Step 1 and Definition 2), Principal Component Analysis (Step 2) to obtain a *small set of Statistically Significant Association Disentangled Spaces DS* (Step 2, Step 3, and Definition 6).

M2, obtain from each DS *AV clusters* (Step 3 Definition 8) and *statistically significant attribute association patterns* (Step 5, Definition 10) and *pattern clusters* (Step 5) without class labels given, or the need for feature engineering and machine training (Step 6 and Step 7)**—**achieving *AV clustering, pattern discovery, pattern clustering* and *entity clustering* in an *All-in-One System*.

M3, use the *intersection of AV Entity ID lists* (Definition 3, Fig. 1c, d) of AVs in an AV Group (Definition 7) from the *Entity ID Address Table* (created at the outset in Step 1, Definition 3) to obtain the *frequency count of AV Groups* for *Frequency-Matrix construction* (Step 1) and *pattern discovery* (Step 5, Fig. 1d)—shifting the search of association or discriminative information or patterns from entities employed in the existing ML models to a direct simple table-lookup and intersecting computation process for pattern discovery and class association—a great *complexity reduction* of the *search process* in ML.

M4, use dataset with class labels to *exploit the ground truth*, and dataset without class label to minimize *label bias* to obtain *auto-error-correcting class associations* (Step5, Step 6 Step 7 and Definition 10) and *entity clusters* (Step 5, Step 6, Step 7 and Definition 11)—an unique way of combining the strength of supervised and unsupervised methods in *auto-correcting class association* and *entity clustering;*

M5, discover *rare and new patterns* from *large* or *small datasets* (e.g., rare implanted pattern [… T R F …] discovered from a group with three entities in Case Study 1), even *imbalanced datasets* (in the thoracic surgical risk experiment in Case Study 5) with *excellent noise tolerance* (synthetic experiment in Case Study 6), since all the patterns discovered have passed three conditions, i.e., they are: (a) obtained from a small set of DS, (b) statistically significant patterns (containing no noise) and (c) statistically disconnected to other AVs or AV groups (disentanglement)—solving the concern of *low volume* and *low quality* data, even *data with new classes* or containing *imbalanced groups*.

M6, produce a Summarized and Comprehensive knowledge bases *interlinking patterns, entities*, and *primary sources* [Step 6 and Step 7], using union patterns for high-level visualization and the comprehensive set of discovered patterns on a small number of statistically significant DS associated with the entities with no information loss—offering *interpretability* and *traceability* to *enhance trust* in ML.

M7, obtain all the above results and knowledge representation within an *All-in-One framework* (Step 6 and Step 7 in the knowledge bases) as exemplified in all experiments reported in the Main text and the Supplementary)—minimizing the *need of method selection and synchronization*.

Using various facets of PDD methodology marked under the header **M** listed above, we will show how PDD has overcome many of the existing limitations raised in machine learning and data mining today. We first list the limitations raised and cite the methodology item(s) used by PDD to overcome them. Then, we will have a better appreciation of the methodology developed as formally defined and explained later. Now, we describe how.

Limitations in Machine Learning and Deep Learning are as follows.

Firstly, in terms of the ethical concerns, bias introduced at any level of development and training causes ethical concerns about ML technology. Using M1 and M2, PDD obtains interpretable patterns associated with primary sources and causes. It identifies and corrects anomalies as well as label discrepancies (M6) to reduce users’ concerns. It relates the interpretable results to organize the knowledge representation and furnish experimental validation, as shown in our case studies and supporting research^9,21,23,24^, for further exploration to gain trust.

Secondly, to reveal cause-and-effect relationship, ML and Deep Learning can make predictions, but not recognize the cause-and-effect relation and explain the reason why they are connected. PDD can provide a compact set of patterns related to distinct sources (M1 and M2) to help interpretation and causal exploration for scientific and further experimental confirmation (M6).

In addition, while neural networks require enormous amounts of high-quality data and intensive training to produce viable results due to entangled patterns and factors in the data, particularly when the dataset is small/imbalanced, PDD discovers disentangled patterns related to specific sources via M1, M2, and M5, and hence is less affected by class size and class imbalance.

Finally, to interpret a decision and a solution is still a challenge in deep learning in certain problem domains. The post-hoc process generates explanations through feature importance-related measures relying on domain-specific knowledge which may only sometimes be available. Deep learning cannot explain the event at a scientific and human-understandable level. Using M1, M2, and M4, PDD can discover compact sets of interpretable patterns associated with the primary sources from the entities for verification in the real world. It uses M4 to interpret inconsistencies, anomalies, and label discrepancies, M5 to identify and interpret rare, new, and imbalanced cases, and M6 for interlinking patterns, entities, and primary sources as to give a full picture of the knowledge discovered.

In machine learning, this is a complex and growing issue exacerbated by a lack of code transparency and model testing methodologies since its throughput is opaque. It affects safety, reliability, and the detection of bias.

PDD uses M1, M2, M4, and M5 to obtain class association results, it (a) does not require training and testing with cross-validation, (b) has great tolerance of noise as shown in our Case Study 6, and (c) can identify and correct label discrepancies disregard wherever they occur. Hence, PDD produces more stable results that help reproductivity.

Challenging problems in Data and Association Rule Mining are as follows.

The first challenge is intensive data collection and system development. Data and association rule mining processes are usually time-consuming, expensive, labor-intensive, and need a large volume of data and extensive post-hoc process to sort out the results.

As shown in M1, M2, M5, and in our case studies, PDD requires fewer data, preprocessing, and postprocessing to produce interpretable summarized/comprehensive knowledge bases with no information loss for visualization and tracking. It efficaciously fulfills the goal of data mining.

The second challenge is the overwhelming amount of rules and patterns are discovered. Due to the redundancy and entanglement of patterns inherent in the data, data and pattern mining models usually output an overwhelming number of patterns and pattern clusters, depleting prediction accuracy and interpretability. In all our case studies, using M1 and M2, PDD renders a compact set of disentangled patterns associated with specific primary sources. It uses union patterns while allowing tracking of all the sub-patterns discovered. In entity clustering, PDD produces only a single visualizable cluster configuration (M5 and 7 in the Supplementary) without fine-tuning.

The final challenge is the issue of accuracy being inferior. Due to the prevalence of redundant and entangled patterns and bias inherent in the data, data mining is not as effective in separating them into classes as deep learning. As shown in Fig. 5, using M1 and M5, PDD obtains accuracy in both class association and clustering comparable to the best of machine learning while furnishing interpretable results. It achieves interpretability without sacrificing prediction accuracy. This is a merit of the all-in-one process.

After giving PDD methodology at a high conceptual level, we will present each step with formal definitions, equations, and explanations.

### Definitions, equations and algorithmic steps

The input dataset, **R**, is an *M×N* matrix, where *M* is the number of entities (i.e., records), *A*^*m*^ = 1,… *M*, each of which is assigned with a unique Entity ID (EID) *i*, and *N* is the number of attributes, *A*_1_, …, *A*_*N*_. As for a mixed-mode dataset containing numerical or categorical values, we discretize the numerical values into quantized intervals with equal frequency such that each attribute would assume a finite set of values treated as categorical values denoted as $A_i = \left\{ {AV_1,AV_2,...AV_j} \right\}$, where *AV*_*j*_ is the *j*th value in *A*_*i*_. For each record, the categorical and discretized values are represented as *A*_*i*_ = *AV*_*j*_ and referred to as discrete attribute values (AVs).

The first step is constructing association matrix with adjusted standardized residuals. Treating an AV pair as a compound event co-occurring on the same entities in the dataset, we intend to find out whether an AV-association is statistically significant. We adopt a well-established statistical measure, known as adjusted statistical residual (SR)^30^ to represent the statistical weight of the event as defined in *Definition 1* for an AV pair, and generalize it for high order AV-association referred to as a pattern if the association is statistically significant.

#### Definition 1. statistical residual (SR)

The statistical residual is a statistical measure, referred to as the adjusted standardized residual, to account for the deviation of the observed frequency of occurrences of a compound event (such as an AV pair) from the expected frequency if the co-occurrence of the event is random (i.e., with statistically independent AVs, Definition 2). For an AV pair (*AV*_1_ ↔ *AV*_2_), it is written as *SR*(*AV*_1_ ↔ *AV*_2_) in Eq. (1)

1 $$\begin{array}{ll}SR\left( {AV_1 \leftrightarrow AV_2} \right) = \frac{{Occ\left( {AV_1 \leftrightarrow AV_2} \right) - Exp\left( {AV_1 \leftrightarrow AV_2} \right)}}{{\sqrt {Exp\left( {AV_1 \leftrightarrow AV_2} \right)} }}\\ \qquad\qquad\qquad\qquad\quad\times \left( {1 - \frac{{Occ(AV_1) \times Occ(AV_2)}}{{T^2}}} \right)\end{array}$$

where *Occ*(*AV*_1_) and *Occ*(*AV*_2_) are the frequency of occurrences of the AV pair where *Occ*(*AV*_1_ ↔ *AV*_2_) is the total number of co-occurrences of the AV pairs (*AV*_1_ and *AV*_2_) in an entity; *Exp*(*AV*_1_ ↔ *AV*_2_) is the expected frequency if the AVs in the pair are independent; and *T* is the total number of records. By converting the frequency of all the distinct AV pairs in the frequency association table obtained from data into SR though the Address Table, we obtain a square SR-Matrix (Definition 3).

#### Definition 2. SR-matrix and AV-vector

An SR-matrix is a matrix of SRs between distinct AVs. Each of its rows corresponding to a distinct AV represents a vector whose coordinates are SRs of that AV associating with other AVs corresponding to the column vector.

Traditional pattern/rule mining searches and confirms significant patterns/item-sets from the relational datasets. Due to possible pattern entanglement, it may take extensive effort to get the significant patterns associated with separate underlying sources. PDD creates the Entity ID Address Table to reduce the algorithmic complexity from *O*(*N*^2^) to *O*(*N*) using a table lookup and a simple set intersection approach to obtain pattern candidates for pattern discovery.

#### Definition 3. Entity ID address table (address table)

It is a table listing, for each distinct AV in the data, the Entity-IDs of the entities embodying that AV.

An example of the Address Table is given in Fig. 1d. It not only provides a direct way to relate AVs contained in each entity but is also used to look for entities containing high-order AV-associations through the intersection of Entity-IDs (*Definition 4*) of the entities containing the AVs.

#### Definition 4. Entity-IDs (EID) intersection

The intersection of the Entity-IDs listed in AVs in an AV-Group in the Address Table represents the EIDs of all the entities containing the AVs in the group.

By this token, PDD obtains the co-occurrence counts of the association from the Address Table instead of from an exhaustive search and counting from the entities in the data. Figure 1d in the Main text gives an example of the Address Table and EID intersection. Such a simple method will ensure statistical robustness and reduce the size/complexity in constructing the association matrix as well as in pattern discovery.

The step 2 is association disentanglement. To obtain projected spaces (axes) of AV-vectors orthogonal to each other, we apply principal component analysis (PCA) on the SR-Matrix. In PDD, PCA is used to extract principal components (PCs) from the SR-Matrix instead of from the original Association Matrix. Hence, the output reflects the statistics of the AV-associations in the disentangled spaces for high-order pattern discovery (Fig. 1g, h).

#### Definition 5. AV-association disentanglement

AV-association disentanglement is a process to obtain the PCs from the SR-Matrix using PCA and then reproject the projections of the AV-vectors on each PC onto a new SR-Matrix referred to as Reconstructed SR-Matrix to render new coordinates of the AV-vectors captured by the PC orthogonal to other PCs.

Figure 1g, h illustrate how the AV-vectors projected onto a PC are mapped onto the Reconstructed SR-Matrix to represent their transformed coordinates in the Disentangled Space DS.

Step 3 is to select statistically significant disentangled space. In PCA, the number of PCs is the same as that of AVs in the SR-Matrix. Since PDD focuses only on statistically significant information, it selects only those disentangled spaces with statistically significant AV-associations to obtain a small set of statistically significant disentangled spaces denoted by DS.

#### Definition 6. Statistically significant AV-association disentangled space (DS)

It is a selected DS such that the maximum SR in its Reconstructed SR-Matrix is greater than the statistical threshold such as 1.96 for 95% confidence interval.

To obtain statistically associated AVs from a DS, PDD generates AV-Group and AV-Subgroups (*Definition 7*) from each DS by AV Clustering (*Definition 8*).

#### Definition 7. Statistically connected AV-Group

An AV-Group is said to be statistically connected if each AV in it must have a significant statistical association with another AV in that group. That is, in that AV-Group, each AV can find at least another AV significantly associated with it.

#### Definition 8. AV clustering

It is a process to group AVs into AV-Groups that contain statistically connected AVs while uncorrelated to other AV-Groups.

Obtaining AV-Groups from DS is a process much superior to feature selection in most ML models. It is automatic, statistically robust, precise, comprehensive, and can be executed in parallel mode. It moves from the feature (attribute) level to the feature (attribute) value level. The pseudo-code of AV Clustering is given in *Algorithm: AV Clustering*.

To get more refined associations of AVs in an AV-Group, we group AVs into AV-subgroups by a similarity measure defined between AVs. First, we introduce the concept of AV cover.

##### Algorithm: AV Clustering

Input: *RSRV*_*k*_, *threshold*

Output: $$AVGs = \left\{ {AVG_1,AVG_2,...} \right\},AVG_i = \left\{ {AV_1,AV_2,...AV_k} \right\}$$

Initial Setting: Randomly add two AVs of the first AV-association in *AVG*_1_

**for** each significant AV association in *RSRV*_*k*_(*RSR*(*AV*_*i*_ ↔ *AV*_*j*_) > *threshold*)

**for** each existed AV-Group

**if** *AV*_*i*_ has been in *AVG*_*l*_: add *AV*_*j*_ in *AVG*_*l*_ end

**end**

**if** both *AV*_*i*_ and *AV*_*j*_ are not in any existed *AVG*_*l*_:

create and new *AVG*_*l*+1_

add both *AV*_*i*_ and *AV*_*j*_ into *AVG*_*l*+1_

**end**

**end**

**Return** *AVGs*

#### Definition 8.1

Let *AV*_*i*_ be an AV. We consider the set of entities containing *AV*_*i*_ as an AV cover of *AV*_*i*_ denoted by *cov*(*AV*_*i*_).

#### Definition 8.2

Let *AV*_*i*_ and *AV*_*j*_ be a pair of AVs. The similarity between them within a data set is defined as the percentage of the cardinality of their EID-intersection over that of their total cover in data (i.e., their union).

2 $$sim\left( {AV_i,AV_j} \right) = \frac{{2|cov(AV_i) \cap cov(AV_j)|}}{{|cov\left( {AV_i} \right)| + |cov\left( {AV_j} \right)|}}$$

For example, in Fig. 1d, the intersection cov(A2 = P) ∩ *cov*(*A3* = *Q*) is the cover of (*A2* = *P*) and (*A3* = *Q*) which reflects the strength of their association between them. If the similarity between two AVs exceeds the lower bound of 50% (by default), they are grouped in the same cluster, otherwise, separated into different clusters.

#### Definition 9. Primary AV-subgroup and primary DS unit

We refer to a set of AVs obtained from an AV-Group as primary AV-subgroups if it is in a DS, statistically connected, with a strong association with other AVs nearby, and uncorrelated to other AV-subgroups. Each AV-subgroup then represents a primary DS unit (DSU) identified by a triple code DSU[#DS #AVG #AVSG] where “#” signifies the ordinal ranking of the item.

As an example, the triple code DSU[1 2 1] signifies that the unit is in DS1, AVG2 and AVSG1.

Since each AV-subgroup obtained is in a DS containing statistically connected AVs, uncorrelated to other AV-Groups, it is presumed to come from a primary source attributed to its occurrence on entities of a certain group such as a taxonomic class or related groups sharing common functionality. By primary, we mean something of key importance—for an individual group or a common functionality shared by several groups. If they are important by themselves, they should be reflected in disentangled AV-associations of individual groups or groups sharing a particular functionality, known or unknown. This is exactly what, why, and how PDD has achieved.

Step 5 is on pattern discovery. After obtaining AV-Subgroups, a pattern discovery procedure is employed to discover high-order patterns (*Definition 10*) from each AV-Subgroup in a DS using a statistical pattern hypothesis testing criterion.

#### Definition 10. High-order pattern

A set of AVs with a size greater than 2 is defined as a high-order pattern if their frequency of occurrences on the same entities in the dataset exceeds the statistical threshold.

**Fig. 6:**
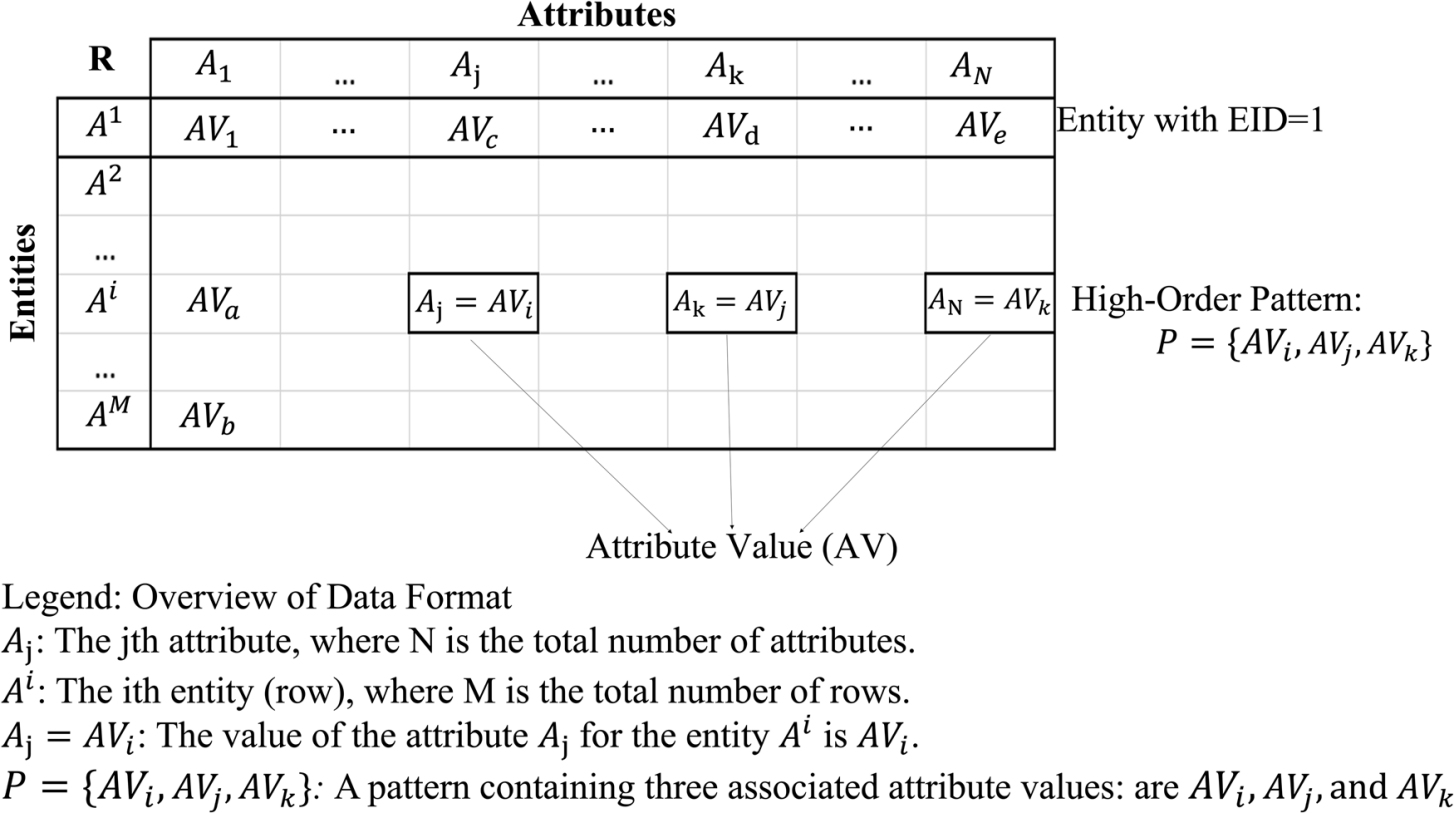
An illustration of an example of the input dataset, EID, and a high order pattern.

Figure 6 gives an example of a high-order pattern. In pattern discovery, taking subsets of AVs in an AV-Groups as pattern candidates, we use the adjusted standardized residual (SR in *Definition 1*) estimated from the frequency of co-occurrences of that subset in the data in a hypothesis test to determine whether it is a statistically significant pattern or not. The SR for a pattern Pj is derived as Eq. (3).

3 $$SR\left( {P_j} \right) = \frac{{R\left( {P_j} \right)}}{{\sqrt {V\left( {P_j} \right)} }}$$

where $\scriptstyle R\left( {P_j} \right) = \frac{{Occ\left( {P_j} \right) - Exp\left( {P_j} \right)}}{{\sqrt {Exp\left( {P_j} \right)} }}$ is the adjusted standardized residual, the *Occ*(*P*_*j*_) is the frequency of the pattern co-occurrences on the same entities; and *Exp*(*P*_*j*_) is the expected occurrences on the same entities, and $\scriptstyle{{{\mathrm{V}}}}\left( {P_j} \right) = 1 - \mathop {\prod}\nolimits_{{{{\boldsymbol{A}}}}^{{{\boldsymbol{m}}}}} {\frac{{Occ\left( {P_j} \right)}}{M}}$ is the standard deviation of all the residuals, M is the total number of entities.

To keep the discovered patterns non-redundant, we only accept delta-closed patterns^31,32^ in the PD process as reported in^33^. In summary, pattern discovery on DS is a process of obtaining high-order patterns from incrementally growing statistically connected AV-subgroups while confirming their pattern status through the hypothesis test^30^.

Step 4 is on entity clustering. To cluster entities, we assign each entity to an AV cluster if the intersection of the cover of that entity and that of the cluster exceeds those with other AV clusters.

#### Definition 11. Entity clustering

It is a process of assigning each entity in the data to an AV-Subgroup by maximizing the number of AVs they share. Considering that an entity contains a set of AVs denoted as *E*_*i*_, and each AV-Subgroup contains a set of AVs. The sharing percentage between them is $\scriptstyle{sharing_{ij}\, = \,\frac{{\left| {E_i \cap AVSG_j} \right|}}{{\left| {AVSG_j} \right|}}}$. The entity is assigned to *EC*_*j*_ if *sharing*_*ij*_ is maximum in the DSU.

Below is the pseudo code of the *Algorithm: Entity Clustering*.

##### Algorithm: Entity Clustering

Input: $$AVSGs = \left\{ {AVSG_1,AVSG_2,...} \right\}$$,

$$Entity = \left\{ {A_1 = AV_1,A_2 = AV_2,...} \right\}$$,

Output: $$ECs = \left\{ {EC_1,EC_2,...} \right\}$$

**for** each entity *E*_*i*_ (which contains a set of specific AVs in the entity)

**for** each *AVSG*_*j*_ (contains a set of associated AVs), *j* = DSU[#PC, #AVG, #AVSG]

$$sharing = \frac{{\left| {E_i \cap AVSG_j} \right|}}{{\left| {AVSG_j} \right|}}$$

**end**

Assign *E*_*i*_ to *EC*_*j*_ by maximizing similarity

**end**

**return** ECs (Entity Clusters)

Steps 6 and 7 are constructing knowledge base and correcting anomaly. PDD obtains interpretable knowledge base and entity clusters with anomaly detection and rectification in a synchronized manner. If classes are given, in the KB, PDD can assign class status to each entity (Step 7) after the anomaly identification and rectification (Step 6).

Since traditional supervised ML models cannot identify anomalies and mislabels at the outset, they usually use k-fold cross-validation via training and testing to randomly distribute the anomalies and entities from uneven classes and data locations for classification evaluation to assist fine-tuning in feature engineering and/or parameter optimization. For PDD, because class associations are unaffected by the location/distributions of the entities, k-fold cross-validation is unnecessary. For PDD, since patterns are obtained from statistically connected AV-Groups in selected DS automatically, a separate feature selection process is unneeded. All our experimental results show that anomalies can be identified, and after discrepancy rectification, PDD can improve the class association and entity clustering results disregarding how the anomalies, noise, and entities are distributed in the data.

In entity class association, PDD meticulously uses the ground truth knowledge from wCL. It then uses the nCL result of entities associated with implicit class labels yet unbiased by them for identifying and rectifying misinformation. We first give the definition of class status and then the Entity Class-Association Rules. A Correctly classified entity is denoted as Cor; a mislabel with Class readjusted as Cra; an outlier as OL; an Incorrectly classified as Inc and an Undecided as Und.

Let *C*_*i*_ be the class given to an entity and *C*_*k*_ is the class of another class *k*. Let *f*_*i*_ and *f*_*k*_ be the frequency of occurrences of the class discovered on an entity pertaining to its given class *i* and another class *k* respectively. Then the class status of an entity is assigned as: Cor if *f*_*i*_ > *f*_*k*_; Inc if *f*_*i*_ < *f*_*k*_; Und if *f*_*i*_ = *f*_*k*_ for certain *k* and *f*_*i*_ is non-zero; OL if *f*_*i*_ = 0 and *f*_*k*_ = 0 for all *k*; and class readjusting (Cra) if *f*_*i*_ = 0, and *f*_*k*_ <> 0 for a certain *k* and the class status is readjusted to *C*_*k*_.

To integrate the results of wCL and nCL, the following *Class Status Integration Rules* are used. (1) An entity is assigned as a Cor, an Inc or a Und if that is found from wCL in the consistency check. They are readjusted to class if found and confirmed from the results in either wCL or nCL. (Rationale: PDD gives strongest weights to the ground truth unless label discrepancy is spotted).

(2) An entity is an Outlier (OL) or an Undecided (Und) if found in both wCL and nCL but assumes the class status as found in either one of them.

To evaluate the class association accuracy of an entity, we use the support (sum of pattern occurrence) of the pattern of its given class over those of the others. The accuracy can be calculated using Eq. (4).

4 $${{{\mathrm{Accuracy}}}} = \frac{{\# Corr + \# Confirmed\;Misclassfied}}{N}$$

The Heart Disease results in Fig. 4a in Main gives several examples for Rule 1. The *cytochrome c* results give a couple examples of Rule 2 (Fig. 3c).

Finally, to render transparent and interpretable results, PDD obtains (1) pattern clusters and entity clusters showing their distinguished pattern and group characteristics, and (2) a succinctly organized and interpretable all-in-one knowledge base linking primary sources, patterns and data together.

If classes are not given, the pattern and entity cluster are the unsupervised outcomes of PDD. Their accuracy can be assessed based on the implicit classes or the unknown primary sources associated with the majority entities possessing the pattern(s) in the DSU. Figure 3c shows that the primary source of DSU groups obtained from nCL correspond closely to those taking taxonomic classes as the primary sources (Fig. 3). When the class is not impacting class association in nCL, PDD discovered the rare group with the pattern [… T Y F…] in the knowledge base and also in the entity cluster (DSU[3 1 1]) in Fig. 3d).

By inter-linking disentangled patterns to individual entities associated with known or unknown primary sources with/without class labels (could be primary sources) as an attribute in the dataset, PDD constructs a comprehensive and a summarized knowledge base and obtains entity clusters simultaneously in support of various ML and clinical tasks without relying on prior knowledge. It contains concise, precise, integrated, explainable, and statistically significant and source related knowledge representation it discovers.

Figures 3c, d and 2c, d in Supplementary together, provides strong evidence expounding the interpretability and traceability of PDD. The Knowledge Space shows the AV-association disentanglement. The pattern Space shows the union and comprehensive patterns discovered in each DSU. The Entity Space shows the disentangled pattern(s) that each entity possesses, relating to the known or unknown primary source. It also displays the class status assigned to each entity and the overall class association accuracy.

### Reporting summary

Further information on research design is available in the Nature Research Reporting Summary linked to this article.

## Supplementary information

Supplementary Material

REPORTING SUMMARY

## Data Availability

The authors declare that the datasets supporting the findings of this study are available within the article and its Supplementary files (Supplementary Data). 1. The heart dataset is availability at from the University of California Irvine Machine Learning Repository: https://archive.ics.uci.edu/ml/datasets/Heart+Disease. 2. The breast cancer dataset is availability at from the University of California Irvine Machine Learning Repository: https://archive.ics.uci.edu/ml/datasets/Breast+Cancer+Wisconsin+%28Original%29. 3. The thoracic dataset is availability at from the University of California Irvine Machine Learning Repository: https://archive.ics.uci.edu/ml/datasets/Thoracic+Surgery+Data.

## Code availability

PDD is accessible via our web server on http://pdd.uwaterloo.ca/Home.
