# Supplementary material for: Theory and rationale of interpretable all-in-one pattern discovery and disentanglement system
Source: NPJ Digit Med. 2023 May 22;6:92. doi: 10.1038/s41746-023-00816-9 (PMC10203344; doi:10.1038/s41746-023-00816-9)
Supplement: Supplementary file 1 — Supplementary Material [file 41746_2023_816_MOESM1_ESM.pdf]

Theory and Rationale of Interpretable All-in-One Pattern Discovery and Disentanglement System

Andrew K.C. Wong[1,*], Pei-Yuan Zhou[1], Annie E.-S. Lee[2]
[1]Systems Design Engineering, University of Waterloo, Waterloo, Ontario, Canada,
[2]Computer Science Department, University of Toronto, Ontario, Canada.
[*]Correspondence: akcwong@uwaterloo.ca

Supplementary: Materials and Additional Experimental Result

In this supplement, we will go through the part briefly described in the Main text and furnish a more comprehensive explanation, exposition, and validation of the key capabilities of PDD, particularly those unique ones as outlined in Table II in the Main Text with greater details.

In the exemplifying presentations of the case studies, we focus on the key capabilities of PDD in 1) creation of PDD Knowledgebase interlinking primary sources, discovered patterns and individual entities — transparent, interpretable, comprehensive yet compact. In the Knowledge Base, we show: a) AV-association disentanglement, b) entity classification with label discrepancy correction synchronized with the transparent entity clustering results, c) discovery of rare groups/cases and classification of imbalanced classes; 2) interpreting and using Knowledge Base to assist further exploration and causal study, such as those on taxonomic and proteomic patterns, cytopathological characteristic of cancerous cells, clinician diagnosis analysis, earlier disease and error detection, clinician decision making and so on. To exemplify PDD's all-in-one framework, we present the results in a common format consisting of a) the problem and data; b) the Knowledge Base, including AV-association disentanglement and class association; c) the transparent entity clustering results synchronized with that of the Knowledge Base; and d) a brief discussion. Since the objective of this paper is to introduce the fundamentals and the unique ideas of PDD, we use a set of case studies with verifiable data and problems of various types to exemplify and validate the

capability of PDD in interpreting and tracking the input, throughput and output of the entire process. We want to show high accuracy, precise interpretability with statistical and functional support from different types of data and problems. As we conjecture that the AV-association Subgroups and DSU found on entities are indeed associated with the known/unknown primary sources, we want to find strong support of the conjecture from the Case Studies backed by established knowledge or new statistical, scientific and/or medical findings. In the meantime, we highlight the performance of PDD and compare it with that of other ML models. At last, we expound in detail the algorithmic platform and process which significantly reduces the time and space complexity and produces a compact explainable knowledge representation for further exploration and decision-making.

In the new PDD paradigm, PDD discovers from DS containing stastical significant disentangled patterns occurring on entities associated with distinct primary sources. Hence it saves considerable effort and time of feature engineering adopted in many existing models. We apply PDD on wCL to take full advantage of the ground truth and discover disentangled patterns from nCL unbiased by Classes for error detection and correction to come up with an Auto-Error-Correcting module to improve class association and entity clustering. Hence, we remove the concerns of the users that the existence of unaware anomalies and label discrepancies in the data may affect the results and decision.

In the new paradigm, when a class label is explicitly given in an entity as an AV, it is considered, and proven in the process, as a primary source associated with the discovered disentangled pattern(s) on the entity. PDD takes it as the ground truth knowledge to obtain class association. We refer to this as the Pattern-Class Association. In the meantime, PDD also obtains results from nCL unaffected by class label to check the consistency of the discovered patterns and the implicit

class label of the entities. When an entity contains pattern(s) of another class instead of the given class, PDD changes the class status and readjust the class label. We call this Class Readjustment and denote the process or the results by Cra. After Cra confirmation, we take the final class status assigned to the entities for performance evaluation.

PDD also discovers rare groups in distinct DS without relying on class labels to obtain new knowledge not given in the ground truth. Hence, in the new paradigm, PDD utilizes the knowledge given in the ground truth and in the meantime identifies and rectifies errors caused by unnoticed biases or label-discrepancies as well as rare groups/classes. The objective of these Case Studies is to use verifiable data and problems to exemplify and validate the conjecture and also the PDD capabilities as listed in Table II.

In traditional ML, since there is no direct way to identify class label discrepancies and rare new classes, outliers and/or biases, imbalanced classes or rare groups or patterns may unnoticedly scatter in the data, we have to rely on k-fold cross-validation via training and testing to randomly distribute the anomalies and/or locate uneven groups in different runs for classifier evaluation. Usually, comparative results are obtained to guide the fine-tuning of the classifier via feature engineering and parameter setting. Hence, it often takes intensive effort of the trainers to screen the data or to use big data to minimize the effect of anomalies. (From here on, we use the term "anomaly" to include class label discrepancies and outliers.) Since PDD uses only statistically connected AVs automatically from DS it does not need feature engineering or search methods. Furthermore, it can identify anomalies from inconsistency check and make corrections if confirmed and discover rare groups and/or imbalance classes wherever they are in the data. Thus, PDD can obtain class-association results from both wCL and nCl and retain only the successful class association patterns/rules for class association. Thus, it can solve small, imbalanced as well

as big data problems. A complete classification and predictive analysis of PDD with Cra will be addressed in a separate paper. In this paper, we will show the efficacy of PDD in detecting anomalies and using Cra to improve entity class-association, expounding its potential in more general predictive analysis.

To further validate PPD capabilities as listed in Table II (Main Text), we use six case studies: two on proteomic data, one on histopathological/cytopathological data, one on clinical data, two on imbalance classes with noise (one on thoracic surgical risk and another on a directly verifiable synthetic data). In each case stduy, we first give a simple description of the dataset and the problem, and then the Knowledge Base obtained. In the Knowledge Base, we display the disentangled patterns possessed by each individual entity as well as its class status attained, including anomaly, outlier and Cra. We then present the unsupervised entity clustering result of the experiment. Finally, we furnish additional explanations elaborating the reasons and efficacy of PDD in achieving its unique tasks. In the Main Text, we use icons in the figures which are easy to follow. In the Supplement, in most of the figures, we retain the format obtained close to the output of the PDD computation program. We will keep the outputs in a more formal manner.

Case Study 1: Cytochrome C

The first example on APC of cytochrome C is described in the Main Text. We use this simple example with distinct biological ground truth to validate every capability listed in Table II and give explanations on how and why PDD can fulfill each task so that it will be much easier to interpret the results of more sophisticated data and problems.

Data and Problem

In bioinformatics, there is a need to identify and analyze local and co-occurring functional sites, elements and regions in bio-sequences. Aligned Pattern Clusters (APC) is an unique way we

developed to discover and locate such regions conserving important functionality within and between bio-sequences with reference [1] [2]. Supplementary Figure 1 shows an example of an APC and how it is obtained [1]. For analytic purposes, we treat an APC as a relational dataset with the aligned sites as attributes and amino acids as AVs. We used this dataset for its distinct taxonomic class, presumed as an important primary source attributed to the conserved amino acid patterns in the functional domain. Such premises can be later verified.

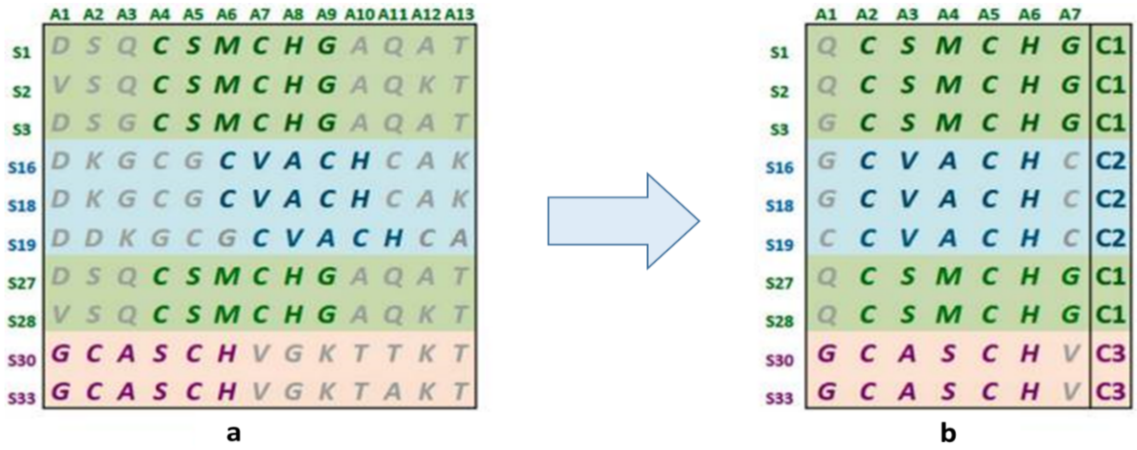

Supplementary Figure 1. Aligned Pattern Cluster APC. **(a).** It shows a portion of protein sequence dataset with embedded high order patterns (in bold). Labels on the top row denote the position in the original sequences and the first column denotes the sequence ID. (**b).** It is an Aligned Pattern Cluster (APC). Based on the presumption that the discovered statistically significant association patterns imply conserved functionality, the amino acids aligned in columns represent functionally/structurally conserved sites to form the aligned pattern cluster revealing the similarity/variation of the functional patterns in this conserved domain of the protein family [1]. Note that after alignment, each site (column) can be treated as an attribute and each item in the column as an AV. The top row gives the AV positions of the patterns in the APC which can be traced back to their location in other settings. The notations C1, C2, and C3 represent the classes (known or unknown at the outset but made known later) of each sequence denoted by the sequence ID.

This dataset contains nine attributes representing nine aligned sites and 92 aligned sub-sequences (containing patterns) taken from taxonomic samples with imbalanced class size (Mammals (MM) 30, Plants (PT) 25, Fungus (FG) 20 and Insects (IN) 7) [1]. We artificially created a rare group by implanting the subpattern [. . . T Y F . . .] on a Mammal, a Plant and a Fungus. That the taxonomic classes are presumed as a primary source is reasonable and verifiable from the results PDD obtained. There could be other primary sources indicating common functionality in the similar

functional domain of different species, particularly in a set of related multiclass data. This example shows PDD can find the primary sources of each class as well as from common functional domains reflected by common sub-pattern(s) for more than one class since both are primary in that functional domain. Such primary sources can be found in a hierarchical and automatic manner based on the intrinsic associations inherent in the data without relying on class labels. This is also a unique capability of PDD.

PDD Knowledge Base (Knowledge Base)

Supplementary Figure 2 represents the compact, yet complete Knowledge Base obtained from the APC of cytochrome c — one set with class labels included in the dataset denoted as wCL, and the other without, denoted as nCL. We use these notations to represent the dataset, or the results obtained from them by PDD. In wCL, class label is treated as an additional AV for finding its association with other AVs. Figure 3(c) in Main and Supplementary Figure 2(a) show the Summarized and the Comprehensive Knowledge Base obtained from wCL respectively. Figure 3(d) in Main and Supplementary 2(b) represent those obtained from nCL. Supplementary Figure 2(c) represents the Knowledge Base obtained from the entities associated with original DSU[1 1 1] which share common subpatterns as shown in DSU[1 1 1] in Figure 3(d) in Main. These few figures entail the essential knowledge discovered by PDD, illustrating the compactness of the all-in-one framework, and validating the key capabilities of PDD.

In this wCL, we selected only patterns containing class labels as an AV to support class association. Thus, we fully utilized the information provided by the ground truth. We called the Knowledge Base a ca-Knowledge Base (stands for Class-Association Knowledge Base). It is just an excerpt of the Knowledge Base of wCL containing patterns with class label as an AV so that we give full weight of the class label given. Later we used the disentangled patterns obtained from nCL,

unbiased by the ground truth (i.e the class label), to spot the inconsistency between the discovered patterns and the implicit class label on each entity. From the inconsistency, we could identify label discrepancy or other misinformation found on the entities, disregarding where they are located, in a small or a big group. They are verifiable because of the transparency and interpretability of the patterns obtained from the Knowledge Base and Entity Clusters. The findings can be related to the source environment in the biology world for further exploration and confirmation. The Summarized Knowledge Base in Figure 3(c) in Main and the Comprehensive Knowledge Base (Supplementary Figure 2(a)) show the superb AV-association disentanglement where pattern(s) discovered in each Disentangled Unit (DSU) occur on entities pertaining to only one class source. The Knowledge Base in PDD consists of a Knowledge Space, a Pattern Space, and an Entity Space. **The Knowledge Space** encompasses the AV-association Disentangled Units (DSUs) and the primary sources as a manifestation of AV-association disentanglement. In each DSU, it displays the number of patterns occurring in the entities associated with a distinct source/class (here, the taxonomic class). A DSU is denoted by three digit code as DSU[#PC, #AV-Group, #AV-Subgroup] where "#" denotes the ordinal ranking of the items DS, AV-Group and AV-Subgroup which represent the Disentangled Space, AV Group obtained in the DS*, and the AV Sub-Group respectively. **The Pattern Space** displays disentangled patterns/pattern-clusters in each DSU. In the Comprehensive Knowledge Base, all the patterns discovered for each entity were displayed. Supplementary Figures 2(a)(b) represent the entire Comprehensive Knowledge Base showing the compact yet comprehensive, succinct, and interpretable output of PDD. In the Summarized Knowledge Base, the union pattern of all the disentangled patterns discovered in each DSU is displayed. It renders an overview of the input (data and known/unknown primary sources), throughput (all the linkages among important elements of the discovery), and the output, a high-

level view of the patterns interlinking with the entities and the primary sources for traceability, providing clues for interactive actions for Q&A NLP platforms. **The Entity Space** lists a) the Entity IDs (EIDs) of all entities with explicit class label displayed in the third row of Supplementary Figures 2 (a)(b) and (c) given in wCL; b) the patterns each possesses by the digit '1' on the EID column and the row linked to its corresponding disentangled pattern in a DSU in the Comprehensive Knowledge Base; and c) the number of patterns (in numeral) each entity possesses in the Summarized Knowledge Base. In these figures, we displayed only a few correctly classified entities with class labels given, but most of the anomalies discovered except a few "Undecided (Und)" in the Fungus (FG) family (Supplementary Figure 2(a)(b)).

a) Knowledge Space Revealing AV-association Disentanglement

In the Knowledge Space from wCL, we found in the primary source columns perfect ***disentanglement*** in both summarized Knowledge Base and Comprehensive Knowledge Base (Figure 3(c) in Main and Supplementary Figure 2(a)). Note that these compact set contains all the disentangled patterns discovered in the dataset. In the wCL, the taxonomic class labels play an important role. As for pattern order (i.e., the number of AVs making up the pattern), in the Summarized Knowledge Base, we displayed only the range of their variations by two notations, e.g., "4*" denotes that the pattern in the DSU is 4 but with variation; while "5_8" implies that it varies from order 5 to order 8.

In the nCL (Figure 3(d) in Main and Supplementary Figure 2(b)), all the DSUs, except DSU[1 1 1], DSU[1 2 1] and DSU[2 1 1], show perfect ***disentanglement.*** They render disentangled patterns with no class label involved yet matching those discovered in the wCL (like DSU[1 1 2], DSU[2 1 1], DSU[2 2 1] and DSU[4 1 1] matching DSU[1 1 2], DSU[2 1 1], DSU[2 2 1] and SDU[4 1 1] in nCL). This indicates that PDD can discover the intrinsic association. In nCL, when class label

is not affecting the pattern association, PDD not only can discover AV-associations associated with the primary sources related to the classes (according to the implicit class labels), but also to those associated with functionality (via sub-pattern(s)) common to more than a single class. For instance, from the first row in the DSU[1 1 1] in the Comprehensive Knowledge Base (Supplementary Figure 2(b)), we observe that it contains a common AV-associations [. . . E E . . .] among Mammal, Fungus and Insect; and a rare case in DSU[3 1 1] (Supplementary Figure 2 (b)) containing the pattern [. . . T Y F …] implanted in a Mammal, a Fungus and an Insect sample. Since each of these samples keeps its class label in wCL, they are not found as a rare group in wCL (Figure3(c) in Main) but found in nCL (Figure3(d) in Main).

Comprehensive Knowledgebase (with class labels)

Knowledge Space							Pattern Space										Entity Space																	
Disentangled Space			Primary Source				Patterns										Entity ID (EID) with implicit class label																	
DS Unit			Pattern Occurrecnce				Attribute Values										Mammal			Plant				Fungus								Inset		
DS	AVG	AVSG	Mammal	Plant	Fungus	Insect	Class	S71	S72	S73	S76	S88	S90	S92	S95	S96	1	31	33	34	58	60	62	63	68	70	71	73	82	84	85	86	91	92
1	1	1	27				Mammal		M	E	E	I		I	K		1		1															
1	1	1	19				Mammal		M	E	E	I	A	I	K		1		1															
1	1	1	13				Mammal		M	E	E	I	A	I	K	G			1															
1	1	2	7				Mammal		M	E	E	I	V	I	K	E																		
1	2	1		22			Plant		Y	D	L	V	P	L	P	Q				1	1		1											
2	1	1	32				Mammal	L	M			I					1		1															
2	1	2		27			Plant	L	Y		L									1	1		1											
2	1	2		27			Plant	L	Y			V								1	1		1											
2	1	2		26			Plant	L	Y				P							1	1		1											
2	1	2		24			Plant	L	Y							Q				1	1		1											
2	1	2		27			Plant	L			L	V								1	1		1											
2	1	2		26			Plant	L			L		P							1	1		1											
2	1	2		24			Plant	L			L					Q				1	1		1											
2	1	2		26			Plant	L				V	P							1	1		1											
2	1	2		25			Plant	L				V				Q				1	1		1											
2	1	2		24			Plant	L					P			Q				1	1		1											
2	1	2		27			Plant		Y		L	V								1	1		1											
2	1	2		26			Plant		Y		L		P							1	1		1											
2	1	2		26			Plant		Y			V	P							1	1		1											
2	1	2		24			Plant		Y			V				Q				1	1		1											
2	1	2		24			Plant		Y				P			Q				1	1		1											
2	1	2		26			Plant				L	V	P							1	1		1											
2	1	2		24			Plant					V	P			Q				1	1		1											
2	2	1			17		Fungus				E	A	G			K									1						1			
2	2	1			6		Fungus	M	S						E	K									1						1			
2	2	1			6		Fungus	M	S			A	G		E	K									1						1			
2	2	1			9		Fungus	M	S		E	A	G			K									1						1			
2	2	1			4		Fungus	M	S		E	A	G		E	K									1						1			
2	2	2			3		Fungus								D															1				
2	2	3			4		Fungus								A																			
4	1	1				7	Insect		F							N																1	1	1
4	1	1				3	Insect		F						A	N																		1

Association Classification Results (with/without class inforamtion)	Entity ID	1	31	33	34	58	60	62	63	68	70	71	73	82	84	85	86	91	92
Perfect pattern disentanglement. Correct = 89 Outlier = 3.	**With Class**	🐈	OL	🐈	🌿	🌿	OL	🌿	🍄	🍄	OL	🍄	OL	OL	🍄	🍄	🐜	🐜	🐜
one found in nCL as Rejusted for Plant																			
**Accuracy:** **Before class readjusting: (92-5)/92 = 94.56%** **After class readjusting: (92-3)/92 = 97.83%**																			

**Legends**				
**Four Classes in the dataset:**	🐈	Mammal	**Class Status for classification:**	Inc In Correct (Misclassified)
	🌿	Plant		OL outlier
	🍄	Fungus		Rare Rare Cases/Entities
	🐜	Insect		Und Undetermined

(a)

Comprehensive Knowledgebase (without class labels)

Knowledge Space							Pattern Space									Entity Space																		
Disentangled Space			Primary Source				Patterns									Entity ID (EID) with implicit class label																		
DSU			Pattern Occurrecnce				Attribute Values									Mammal			Plant				Fungus								Inset			
DS	AVG	AVSG	Mammal	Plant	Fungus	Insect	S71	S72	S73	S76	S88	S90	S92	S95	S96	1	31	33	34	58	60	62	63	68	70	71	73	82	84	85	86	91	92	
1	1	1	32		6	7			E	E						1		1								1					1	1	1	🐕
1	1	1	24			4			E	E	I	A				1		1													1	1		
1	1	1	27					M	E	E	I		I	K		1		1																
1	1	1	19					M	E	E	I	A	I	K		1		1																
1	1	1	13					M	E	E	I	A	I	K	G			1																
1	1	2	7					M	E	E	I	V	I	K	E																			
2	1	1	32				L	M			I					1		1																
1	2	1		22	1			Y	D	L	V	P	L	P	Q				1	1		1					1							🌿
2	1	2		24	1		L					P			Q				1	1		1					1							
2	1	2		24	1			Y				P			Q				1	1		1					1							
2	1	2		24	1					L		P			Q				1	1		1					1							
2	1	2		24	1						V	P			Q				1	1		1					1							
2	1	2		25	1		L				V				Q				1	1		1					1							
2	1	2		24	1			Y			V				Q				1	1		1					1							
2	1	2		24	1					L	V				Q				1	1		1					1							
2	1	2		26	1		L				V	P							1	1		1					1							
2	1	2		26	1			Y			V	P							1	1		1					1							
2	1	2		26	1					L	V	P							1	1		1					1							
2	1	2		24	1		L	Y							Q				1	1		1					1							
2	1	2		24	1			Y		L					Q				1	1		1					1							
2	1	2		26	1		L	Y				P							1	1		1					1							
2	1	2		26	1			Y		L		P							1	1		1					1							
2	1	2		27	1		L	Y			V								1	1		1					1							
2	1	2		27	1			Y		L	V								1	1		1					1							
2	1	2		24	1		L			L					Q				1	1		1					1							
2	1	2		26	1		L			L		P							1	1		1					1							
2	1	2		27	1		L			L	V								1	1		1					1							
2	1	2		27	1		L	Y		L									1	1		1					1							
2	2	1			19						A	G			K								1	1		1				1				🍄
2	2	1			11		M	S			A	G			K								1							1				
2	2	1			6		M	S			A	G		E	K								1							1				
3	1	1	1	1	1				T	Y	F						1				1								1					Rare
4	1	1				7		F							N																1	1	1	🐜

DS: Disentangled Space; AVG: AV-Group; AVSG: AV-Subgroup

Results:	Entity ID	1	31	33	34	58	60	62	63	68	70	71	73	82	84	85	86	91	92
	Class Status Assigned	🐕	Rare	🐕	🌿	🌿	Rare	🌿	🍄	🍄	OL	Und	🍄	OL	Rare	🍄	Inc	Inc	Und

Applying PDD to entities associated with DSU[1 1 1] rendered disentangled paterns assocatied with classes.

PDD found the rare group with implanted pattern [...T Y F ...] in a Mammal, a Plant and a Fungus.

Legends

Four Classes in the dataset: 🐕 Mammal; 🌿 Plant; 🍄 Fungus; 🐜 Insect

Class Status for classification: Inc In Correct (Misclassified); OL outlier; Rare Rare Cases/Entities; Und Undetermined

(b)

Summarized Knowledgebase for DSU [1 1 1] (without class labels)

Knowledge Space							Pattern Space									Entity Space													
Disentangled Space			Primary Source				Patterns									Entity ID (EID) with implicit class label													
DSU			Pattern Occurrecnce				Attribute Values																						
DS	AVG	AVSG	Mammal	Plant	Fungus	Insect	S71	S72	S73	S76	S88	S90	S92	S95	S96	1	4	5	15	25	26	31	33	34	35	39	40	45	
2	1	1	26					M					I	K		1							1						🐕
2	2	1			1	5		F					L		N											1	1	1	🐜
2	2	1			3	2		F					L	P												1	1		🌿
2	2	1			1	3		F					L	A														1	🐜
2	2	1			1	2		F						P	N											1	1		🐜
2	2	1		1	5						A	G	L		K									1	1				🌿
2	2	1				3		F			V		L	A	N													1	🐜
2	2	1		1	4			F			A	G	L		K									1					🌿
3	1	1				3		F			V			A	N													1	🐜

Results:	Entity ID	1	4	5	15	25	26	31	33	34	35	39	40	45
	Class Status Assigned	🐕	OL	OL	OL	OL	OL	OL	🐕	Inc	🌿	🌿	🐜	🐜

Attribute Value Associations (from DSU [1 1 1]) are disentalged into distinct primary sources corresponding to taxonomic classes.

DSU[2 2 1] can easily be clustered into two subgroups. Then the disentalgement is superb.

Legends

Four Classes in the dataset: 🐕 Mammal; 🌿 Plant; 🍄 Fungus; 🐜 Insect

Class Status for classification: Inc In Correct (Misclassified); OL outlier; Rare Rare Cases/Entities; Und Undetermined

(c)

Supplementary Figure 2. Complete Results from Applying PDD to APC of Cytochrome c. These figures represent the complete output of PDD when applied to an Aligned Pattern Cluster obtained from cytochrome c protein family [1] with taxonomic classes: Mammal (MM), Plant (PT), Fungus (FG) and Insect (IN). It includes the PDD Knowledge Base (Knowledge Base) and the Entity Clusters. **(a)** and **(b)** are the Comprehensive Knowledge Base obtained from the relational dataset with and without class label given respectively. **(c).** It shows further

disentanglement on the entities found in the Disentangle Space Unit DSU[1 1 1] obtained from the dataset with no class labels given.

For a multiple class problem like this one, certain primary sources of entities in a DSU like DSU[1 1 1] could come from the common functionality among classes, but their primary sources related to distinct classes can still be found in some disentangled space or via further disentanglement (Supplementary Figure 2(c)). This case study shows the unique hierarchical disentanglement capability of PDD which can disentangle patterns from entities associated with distinct classes as well as common functionality among classes automatically without relying on outside clues/guides. It supports our premises that primary source implies something of functional importance to entities associated with specific AV-associations/patterns discovered by PDD. This is what PDD has revealed in both the cytochrome c and class A scavenger receptor data. Such revelation provides interpretability and guidance for further exploration.

EID84 is an interesting case. It contains a second order pattern of [FG … A] when Fungus is the class label and A occurs only in Fungus and Insect. It contains no other pattern. Hence in wCL it is classified as FG. However, in nCL where no class label exists, it is found to pertain to the group with the implanted rare pattern. Then our implantation is the primary source. Hence EID84 is both a Fungus and contains a pattern from another source related to the implanted rare pattern. PDD is able to offer an in-depth analysis of this case.

b) Pattern Space for Pattern Analysis and Interpretability

In the Summarized Knowledge Base, the union pattern in a DSU is represented by the union of the disentangled patterns in that DSU stored in the Comprehensive Knowledge Base. It provides a succinct and compact way to offer a pattern overview in the DSU while a complete set of patterns of each entity can be retrieved from the Comprehensive Knowledge Base right away. From the wCL (Supplementary Figure 2(a)), we could see various pattern(s) associated with only a single

class label as its primary source. Patterns containing class labels as an AV can serve as patterns/rules for class association. From nCL (Supplementary Figure 2(b)), succinct patterns at a certain source level cannot be related to the primary source at class label level when entangled in entities without AV-association disentanglement. However, they can be revealed by further disentanglement (Supplement Figure 2(c)).

c) Entity Space for Class/Group Association Supporting Anomaly Detection and Correction. In the entity space of the Comprehensive Knowledge Base (Supplementary Figure 2 (a)), PDD gives each entity an EID, a class icon of its explicit or implicit class label and the pattern(s) it possesses in the pattern space. For an entity, the digit "1" on a row intersecting with the entity EID Column denotes the pattern(s) in the pattern space the entity possesses. For example, in the Comprehensive Knowledge Base (Supplementary Figure 2(a)), E1 contains the patterns in the first two rows of the Pattern Space (from here on, to simplify the presentation we will use E1 instead of EID1 and so forth). In the Summarized Knowledge Base, the numeral "2" in DSU[1 1 1] denotes the number of patterns in the DSU possessed by the entity as displayed in the Comprehensive Knowledge Base. Furthermore, from the digit(s) of the patterns each entity possesses on its EID column, PDD can check the consistency of its pattern(s) with its explicit or implicit class label by the following rules.

An entity pertains to

1. Cor (stands for correctly classified) if a pattern discovered in compliance with its given class label has the "support" (i.e., the total number of patterns of a class label that the entity possesses) exceeding those of other classes.
2. Inc (incorrect classified) if otherwise.
3. OL if it contains no statistically significant patterns.

4. Cra if it possesses no pattern of its explicit or implicit class label but that of another class with its class label readjusted to the confirmed class.
5. Und (undecided) if the entity has equal support from different classes.

We should note that while the pattern(s) of an entity obtained in wCL is (are) directly associated with the given class label, it shows strong support of being classified into that class unless its class label is questionable. If the entity is found possessing a pattern of another class and none of its given class, it is an Cra with its class label readjusted. Often, if mislabels or biases exist in wCL, without the influence of class label, PPD can discover the inconsistency more effectively in nCL. PDD then integrates the results from wCL and nCL to fully exploit the given knowledge while in the meantime providing a consistency check to remove or correct the possible bias unnoticed in the wCL. The adjustment results are shown in the last (final) EID row in the result section of the wCL (Figure 3(c) in Main).

Here we will give examples of how PDD conducts the pattern-class consistency check on nCL where the AV-associations were not influenced by class labels. In Figure 3(c) in Main, we observe that E31 was given an explicit class label as Mammal and was found as an OL in wCL, whereas in nCL, PDD found that E31 pertains to a rare group containing the implanted pattern […T Y F . . .] with two other entities E60 and E84 with class label Fungus and Insect respectively. This cluster was not found in wCL because each of its members is constrained by its originally given class label but found in nCL when the class labels were absent. This exemplifies the case when class label influence is removed, the rare subgroup not influenced by class label is found. In another example, E73 was given a class label as Fungus. However, it was found in wCL as an OL whereas in nCL as a Plant. That was confirmed in Knowledge Base and Entity Clusters as possessing only Plant pattern(s). Therefore, to discover an unnamed or misnamed rare group or class label

discrepancies, an unsupervised method is necessary to work with the supervised method as we propose in the all-in-one PDD system. This case study shows such and other capabilities of PDD listed in Table II {1-3, 5-12}.

Entity Clustering

Without specifying the number of clusters or setting any optimization criterion to direct the clustering, PDD obtained six clusters from DSUs (Figure 3(e) in Main) based on the disentangled patterns discovered. They were naturally separated as they all came from statistically significant AV-association disentangled spaces DSs. Since these entity clusters were obtained from hierarchical clustering based on the degree of AVs shared by entity pairs, we observed some variation but not crucial, e.g. PDD found two Mammal clusters in DSU[1 1 1] and DSU[1 1 2] automatically and revealed their difference in S90 and S96 (Figure 3(c) in Main).

Discussion

As PDD automatically corrects the class label discrepancies, it naturally places the corrected ones to their right cluster based on the disentangled patterns they possess in the DSU, disregarding what class label is given or discovered. For example, in this experiment, E73 was labeled as a Fungus but discovered as a Plant and being placed into the Plant cluster. Hence, it was marked as a Cra and considered as correctly placed. However, E70 was labeled as an Fungus but found as an OL in Knowledge Base and placed into the Insect cluster. We consider it misplaced. As shown in the Summary at the bottom of the table, PDD obtained an accuracy of 97.82% before Cra and 98.91% after.

After organization and display of the transparent Knowledge Base, PDD also made the entity clustering transparent (Figure 3(e) in Main) as it automatically partitioned the entities into eight clusters from the DSUs and revealed the disentangled patterns/pattern-clusters on each. Finally,

we noted that all the information in the Knowledge Base and Entity Clusters, including anomaly detection and correction, were obtained by PDD on nCL with class label readjusted from the integrated results of wCL— an all-in-one process. They were automatically synchronized.

In this dataset, using taxonomic class as presumed primary sources, PDD obtained consistent and unifying results even for such a small dataset. It not only discovered patterns associated with primary source, such as the taxonomic classes, but also discovered sources associated with the functionality common to different groups such as to Mammal, Fungus and Insect in DSU[1 1 1] and to Plants and Fungus in DSU[2 1 2] (Supplementary Figure 2(b)). The results of all these tasks exemplify and validate PDD capabilities as listed in Table II {4-12}. Although the tasks unique to what PDD achieved are not the same as achieved by other ML models, the high accuracy of class association and entity clustering can match results of the best of the existing methods (Figure 5 in the Main). In the meantime, all the results obtained are explainable and traceable. Therefore, they can be used for exploratory study jointly with other scientific methods. PDD has a unique role to play in providing statistical support and explainable insights.

Case Study 2: Class A Scavenger Receptor APC

Data and Problems

In the second case study, we used another verifiable dataset of APC obtained from the Class A Scavenger Receptors (SR-A) with amino acids as distinct AVs and the subclasses [1] [3] as possible primary sources. SR-A is a diverse family of proteins characterized by their ability to bind modified lipoproteins [3]. Although the 5 members (*Marco, SRA, Scara3, Scara4, Scara5*) [1] [3] of this family could bind modified lipoproteins, they are different in terms of their sequence pattern, location, structure, and therefore function (Supplementary Figure 5). For instance, within the same family, their protein length varies from 451 to 732 with the functional domains residing in different

sequence locations (Supplementary Figure 5). Thus, SR-A is a protein family, with conserved yet diverse function subgroups, ideal in using it to validate the capabilities of PDD in handling multi-classes and relating the findings to the proteomic real world.

The APC of SR-A contains 95 samples and 12 attributes [1] [3]. This receptor has five distinct classes (*Marco, Sra, Scara3, Scara4,* and *Scara5*) located in five different function domains: Cytoplasmic, Collagenous, Transmembrane, a-helical and coiled-coil motifs (Supplementary Figure 4(b)). Since obtaining subclass classification and locating functional domains become important in proteomic study and fighting disease, this dataset was used to test whether PDD can fulfill such demand.

PDD Knowledge Base

Supplementary Figure 3 gives the Summarized Knowledge Base obtained from the APC of SR-A and a subset associated with DSU[1 1 1]. Since SR-A is a multi-class problem, we would like to use this case study to show how PDD handles it. As we shall see, the information from both Knowledge Bases and Entity Clusters are synchronized from the same run with interpretable results fulfilling the all-in-one capability of PDD as listed in Table II.

a) Knowledge Space and Pattern Space showing AV-association Disentanglement. In this dataset with five sub-classes, we found that PDD obtained disentangled DSUs associated with individual classes as well as classes sharing sub-patterns. Supplementary Figure 3(a) is the Summarized Knowledge Base obtained from wCL containing class label as an AV. To disentangle AV-associations from five subclasses, PDD obtained 25 DSUs altogether. Among these, 17 rows (with class color-code) of them associated with a single distinct subclass; and 9 rows found in entities associated with more than one class. Note that two DSUs (DSU[3 1 1], DSU[8 1 1]) associated with all subclasses (revealing pattern common to them all); three with the group *Marco,*

Scara5, and *Sra* (DSU[1 1 1], DSU[2 2 1] and DSU[9 1 1]); one with *Scara4, Scara5* and *Sra*; one with *Marco* and *Scara3* DSU[4 1 1]; one with *Marco* and *Scara5*, DSU[10 1 1] and one with *Scara4* and *Sra* DSU[11 1 1] as the governing class label. For a multiple class dataset of subclasses of a same family, this result is not a surprise since there should be common subpattern(s) within the family. Yet, from the Knowledge Base, we also found patterns associated with primary sources from distinct subclasses (Supplementary Figure 3(b)). When we applied PDD on each group sharing common patterns in the Knowledge Base, we obtained disentangled subpattern(s) associated with distinct subclasses again. Supplementary Figure 3(c) and (d) illustrate two of such groups, the *Scara3* and *Scara4* group and the *Marco*, *Scara5* and *Sra* group respectively. Supplementary Figure 3 (c) shows disentangled pattern(s) associated with distinct subclasses as well as with certain groups. Without relying on prior knowledge, PDD disentangles AV-associations unveiling the intrinsic primary sources when they have subpattern(s) common to each other. Supplementary Figure 3(b) shows the abridged caKnowledge Base consisting only with patterns associated with distinct classes extracted from the Knowledge Base (Supplementary Figure 3(a)). It was used to assign class status to the entities based on the class association rules (in Methodology) with results shown in the summary section in the Entity Space (Supplementary Figure 3(b)).

b) Entity Space: Class Association, Rare Group and Anomaly Detection and Rectification. In the Entity Space of the Summarized Knowledge Base from caKnowledge Base (Supplementary Figure 3(b)), the third row displays the class color-code of each EID. For example, E1 of *Marco* is shown by an orange-code. And, to each DSU, we assigned the class-color code of the primary source (here the taxonomic class given) associated with the majority of its entities. For instance, DSU[1 2 1] was assigned a red-code of *Scara4* since the disentangle patterns discovered in it occur

(column 6 in the Knowledge Space) in entities (such as the representative E57) associated mainly with sacar4. For DSUs associated with more than one group, we left them blank (e.g. DSU[1 1 1]) (Supplementary Figure 3(a) in the original Knowledge Base from wCL).

Summarized KB wCL Containing explicit CL in the RDS

Knowledge Space									Pattern Space													Entity Space																		
DS* DSU			Primary Source Pattern Occurrecnce					Statistics	Patterns AVs													EID with explicit class label Originally given																		
PC	AVG	AVSG	mrco	scara3	scara4	scara5	sra	Order	Class	A234	A235	A236	A237	A238	A239	A240	A241	A242	A243	A244	A245	1	20	21	22	33	34	57	58	65	74	78	79	85	92	94	95	96	97	98
1	1	1	114			112	58	2_7	marco	C	R	M			F	S	G	G		A	V/L	7	4						6	6	6	6	6	3			6			
1	1	2				21		4	scara5	C	R	M																	1	1	1	1								
1	2	1			456			9	scara4	V	A	I			Y	K	V	V	E	K	M						24													
2	1	1		180				3_9	scara3	S	I/L				T	T	D	L	L		E			20		20														
2	2	1	3			229	15	3		C	R										V								1	1	1	1	1	1			1			
3	1	1	229	13	24	21	17	2					L	G								1	1	1	1	1	1	1	1	1	1	1	1	1	1	1	1			
3	2	1		9				3							T	T					E			1		1														
4	1	1	129	12				7	marco							S	R	G/Q	R	A/I	L/S	9	10																	
4	1	2		3				7		Q						S	S	Q	R	I	S																			
5	1	1		3				Q								Q		I	S	0	3						1													
6	1	1		3				Q								Q		I	S	0	3						1													
7	1	1				21		2	scara5			M																	1	1	1	1								
7	2	1					33	5	sra			S			Y	Q			Q	N													2	1			2			
7	2	2					3	3	sra			S								T																				
8	1	1	20	13	24	21	17	2					L	G								1	1	1	1	1	1	1	1	1	1	1	1	1	1	1	1			
9	1	1	20			21	15	2		C	R											1	1						1	1	1	1	1	1			1			
9	2	1			22		1	2		V	A																1	1							1					
10	1	1	20			21		3		C	R	M										1	1						1	1	1	1								
11	1	1			24	1	15	2							Y			V									1	1					1	1		1	1			
12	1	1	17					2							Y			G				1	1																	
13	1	1			24			2	scara4								V										1	1												
14	1	1	20					2	marco							S						1	1																	
15	1	1				19		2	scara5											E									1		1	1								
16	1	1	14					2	marco											A		1																		
17	1	1					15	2	sra			S																					1	1			1			

(a)

Class Association KB (caKB) wCL

Knowledge Space									Pattern Space													Entity Space																		
DS* DSU			Primary Source Pattern Occurrecnce					Statistics	Patterns AVs													EID with explicit class label Originally given																		
PC	AVG	AVSG	mrco	scara3	scara4	scara5	sra	Order	Class	A234	A235	A236	A237	A238	A239	A240	A241	A242	A243	A244	A245	1	20	21	22	33	34	57	58	65	74	78	79	85	92	94	95	96	97	98
12	1	1	17					2							Y			G				1	1																	
14	1	1	20					2	marco							S						1	1																	
16	1	1	14					2	marco											A		1																		
2	1	1		180				3_9	scara3	S	I/L				T	T	D	L	L		E			20		20														
3	2	1		9				3							T	T					E			1		1														
4	1	2		3				7		Q						S	S	Q	R	I	S																			
5	1	1		3				Q								Q		I	S	0	3						1													
6	1	1		3				Q								Q		I	S	0	3						1													
1	2	1			456			9	scara4	V	A	I			Y	K	V	V	E	K	M						24													
9	2	1			22		1	2		V	A																1	1							1					
13	1	1			24			2	scara4								V										1	1												
1	1	2				21		4	scara5	C	R	M																	1	1	1	1								
7	1	1				21		2	scara5			M																	1	1	1	1								
15	1	1				19		2	scara5											E									1		1	1								
7	2	1					33	5	sra			S			Y	Q			Q	N													2	1			2			
7	2	2					3	3	sra			S								T																				
17	1	1					15	2	sra			S																					1	1			1			
																				wCL		1	20	21	22	33	34	57	58	65	74	78	79	85	92	94	95	96	97	98
																	CL Results			nCL		1	20	21	22	33	34	57	58	65	74	78	79	85	92	94	95	96	97	98
																				Final		1	20	21	22	33	34	57	58	65	74	78	79	85	92	94	95	96	97	98

Summary

Total 98 Final CL marco 20 scara3 13 scara4 24 scara5 21 sra 17

Rare 3 Cra for sra to scara4 = 1 OL= 0 Und= 2

Marco scara3 scara4 scara5 Sra Cra scara4 OL Und

Class Association Accuracy: 3 implanted OLs were not counted Total = 95. Before Cra: 2 OLs and 1 mislabeled (E92) Accuracy = (95-3)/95 = 96.84% Class colour-code for different class status

After Cra : 0 OL, 2 Und and 1 Cra (E92) Accuracy = (95-2)/95 = 97.89%

(b)

PDDKB for marco, scara5 and sra KB

DSU			Primary Sources		Patterns													Entities								
DS	AVG	AVSG	scara3	scara4	Class	A234	A235	A236	A237	A238	A239	A240	A241	A242	A243	A244	A245	1	2	7	13	14	31	32	33	37
1	1	1	288		scara3	S	I/L	M			T	T	D	L	L	R	E	38			38					
1	2	1		381	scara4	V	A	I			Y	K	V	V	E	K	M					20	20			
2	1	1	3			Q						S	S	Q	R	I	S			1						
3	1	1		3				V							Q										1	1
																	CL after Cra	1	2	7	13	14	31	32	33	37

(c)

PDDKB for marco, scara5 and sra nCL

DSU			Primary Soource			Patterns													Entites									
DS	AVG	AVSG	marco	scara5	sra	Class	A234	A235	A236	A237	A238	A239	A240	A241	A242	A243	A244	A245	1	16	20	21	29	35	41	42	55	58
1	1	1	20	21	15		C	R											1	1	1	1	1	1	1	1		1
2	1	1	139		6	marco						Y	S	R	G	R	A	L	15		4					1		1
2	1	2	3			marco							S	K	G				1									
2	1	3	4			marco							S		G		G				1							
2	2	1		80	38	scara5						F	R	G	V	E	E	V				6		5	6	3		3
3	1	1		27		scara5			M			F	R			E	E					2			2			
3	2	1			31	sra			S			Y	Q		V	Q	N									2		2
3	2	2			3	sra			S								T											
4	1	1	20	21	15		C	R											1	1	1	1	1	1	1	1		1
													CL after Cra						1	16	20	21	29	35	41	42	55	58

(d)

Supplementary Figure 3. Knowledge Base Results from Class A Scavenger Receptor (SRA) Relational Dataset. (a). This is the summarized Knowlsge Base. Its Knowledge Space shows the AV-association disentanglement related to the primary sources according to the common functionality shared by subclasses as well as by each individual class. For instance, in DSU[2 2 1] contains a pattern [C R . . . V] and DSU[9 1 1] contain the pattern [C R . . .]. DSU[3 1 1] contains the pattern [. . . L G . .] common to all. This shows the succinct revealing capability of PDD for a multiple classes problem. Later we shall see in 3(c) and (d) that the primary sources related to each individual class of these groups of entities can also be found. As in other DSUs, the Pattern and Entity Space display disentanglement pertaining to distinct classes. **(b).** This is the summarized knowledge base displaying only patterns containing class labels. It is extracted from that in Supplementary Figure 3(a) containing patterns embodying explicit class labels as an AV to obtain entity class association. We also call it Class Association Knowledge Base (caKnowledge Base). It exploits the given class label in entity class association. However, it could be biased by the given class label as well. Hence, after assigning the class status to each entity, we use the results obtained from nCL not affected by the class label for consistency check to identify and readjust the entities (Cra) where label discrepancies were identified and confirmed. We displayed the nCL results in the second EID row at the bottom section of Supplementary Figure 3(b) and the final integrated class status as shown in the last row marked Final. After integration, we found 2 Und (E22 and E94) and 1 Cra (E92) coming up with a class association accuracy of 96.84% before Cra and 97.89% after. **(c) and (d).** The summarized knowledge base for sub-class ***Scara3*** and ***Scara4*****,** and ***Marco*****,** ***Scara5*** and ***Sra*****.** These two figures show that patterns entangled due to common functionality among subclasses can be further disentangled to reveal distinct primary sources representing distinct classes.

For class association, we used patterns in the Comprehensive caKnowledge Base only where each pattern is associated with a distinct class. The last EID row in the bottom Summary Section (Supplementary Figure 3(b)) shows the final class status obtained by PDD after integrating the results of the first and the second row obtained from caKnowledge Base, and Knowledge Base from nCL. Here, we notice that most of the entities are correctly classified as their discovered class label complies with the given class label (with the same light color-code) in the EID row above (the fourth row in the Entity Space). PDD also discovered 3 implanted OLs (E96, E97, E98). We considered them as correct but did not use them in the accuracy estimation. Thus, we took 95 as the total and considered 3 other OLs as unclassified to come up with an accuracy as 96.84%. The consistency check found out that among the 2 other OLs found in wCL, one (E92) was found to be a Cra (for it was found containing a pattern of *Scara4* instead), confirmed also in entity clusters

(Supplementary Figure 4); and another (denoted as common) was found containing common patterns for all classes. Hence the accuracy after Cra is 93/95= 97.89%. This experiment shows that PDD can discover class status of entities with multiple subclasses, a small OL group and Cra to improve the class association. The patterns in caKnowledge Base can be used as rules for a supervised classifier using the same approach of anomaly removal and will be reported in another paper.

Entity Clustering Results

Supplementary Figure 4 displays PDD entity clustering results synchronized with Knowledge Base in the same run. In ML clinical applications today, to obtain synchronized results from different procedures is still a challenge. By synchronization, it does not mean the results are identical. It implies that they are derived from the same algorithmic process. For this five subclasses problem, PDD obtained eight clusters, one for *Marco*, two for *Scara3*, one for *Scara4*, two for *Sra* and one consisting only two samples (one of *Scara3* and the other *Sra*, corrected as *Scara4*). The *Scara3* was also found as an outlier. As this dataset with multiple subclasses shown, in the new PDD paradigm, we do not rely on multi-objective optimization but obtain results through automatic and natural disentanglement of statistics distributions presumed to be governed by primary sources (the driver of the underlying AV-associations, such as functionality/taxonomy). Here we observe that sub-clusters are automatically formed based on inherent differences of the AV-association patterns. To cite an example, samples of *Scara5* (C2 and C8 in blue code) are broken into two subgroups. It is due to the differences of the AVs in A240, A241, and A242 in a small group of three entities (E63, 66, 76). This indicates that PDD can identify and locate mutants fast — an important step in genomic/proteomic research.

To give a fuller picture of the cluster, Column 1 of Supplementary Figure 4 show the associated class of each cluster based on the implicit class label of its majority member in the distinct DSU in the Knowledge Base of nCL. Here we found from nCL (not shown in the paper) five OLs (E22, 92, 94, 96, 97, 98). The first two were found in wCL as Sra (Column 6 Supplementary Figure 4). The consistency check found 3 of them were implanted and so did not include them as misclassified. One (E92) listed as *Sra* contained only *Scara4* patterns. So, it was considered as a Cra. but not being placed into any known cluster. It was considered misplaced. Another one (in blank), referred to as "common", contained a common sub-pattern shared by all subclasses (in blank). Hence the total number of misplacements came up to three, giving us an accuracy of 92/95= 96.84% very close to that obtained for Knowledge Base (Supplementary Figure 3(a)). The synchronized results obtained from the Knowledge Base and Entity Clusters of SR-A APC data together with that from Cytochrome C APC, validate PDD's capabilities as listed in Table II for solving multiple class problems.

The comparison results in Supplementary Figure 4(b) shows that PDD outperformed K-Means significantly in all scores. Tracking back to the clustering results [4], we found that K-means could not separate *Marco* from *Scara5* and *Sra* based just on similarity since they are in the same collagenous domain. However, PDD revealed their commonality in DSU[1 1 1] but clearly separated them as shown in Supplementary Figure 4(a) --- DSU[1 1 1], DSU[14 1 1] and DSU[16 1 1] for *Marco,* DSU[1 1 2], DSU[7 1 1], DSU[15 1 1] for *Scara5* and DSU[7 2 1], DSU[7 2 2] and DSU[17 1 1] for *Sra.* Note that each DSU also revealed the unique characteristics of each subclass in the Pattern Space. The unsupervised results in the Entity Clusters also separated them as distinct clusters (C1 for *Marco,* C3 and C8 for *Scara5,* and C9 for *Sra.*) Though the comparative results are impressive, they are still based on the ground truth without anomaly correction. After

that, we found that only two entities among 92 were misplaced. PDD attained an accuracy of (95-2)/95=97.89%. Overall, the results validate the key capabilities of PDD in a superb way --- robust, transparent, and accurate, fulfilling what is proposed in the new paradigm.

Cluster #		DSU			Classes		AVs												Entity
Group Size	EID	DS	AVG	AVSG	Implict	Discovered	A234	A235	A236	A237	A238	A239	A240	A241	A242	A243	A244	A245	Placement
C1 23	1	1	1	1	marco	marco	C	R	M	L	G	Y	S	K	G	R	A	L	Cor
marco 20	20	1	1	1	marco	marco	C	R	M	L	G	Y	S	S	G	R	G	L	COr
scara5 2	65	1	1	1	scara5	scara5	C	R	M	L	G	F	P	G	A	E	D	V	Misplaced
sra 1	74	1	1	1	scara5	scara5	C	R	M	L	G	F	K	G	A	E	E	V	Misplaced
	92	1	1	1	Sra	Und	C	K	M	L	G	Y	T	G	V	A	Q	V	Misplaced
C2 3	63	1	1	2	scara5	scara5	C	R	M	L	G	F	H	S	V	E	E	V	Cor
scara5 3	66	1	1	2	scara5	scara5	C	R	M	L	G	F	P	S	A	E	D	V	Cor
	76	1	1	2	scara5	scara5	C	R	M	L	G	F	R	G	A	K	E	I	Cor
C3 26	34	1	2	1	scara4	scara4	V	A	I	L	G	Y	K	V	V	E	K	M	Cor
scara4 24	57	1	2	1	scara4	scara4	V	A	V	L	G	Y	K	V	V	Q	R	V	Cor
	96	1	2	1	outlier	outlier	V	L	I	G	L	L	L	E	R	D	G	S	N/A
	98	1	2	1	outlier	outlier	A	G	Q	D	T	L	E	I	A	E	Q	I	N/A
C4 10	21	2	1	1	scara3	scara3	S	I	M	L	G	T	T	D	L	L	R	E	Cor
scara3 9	33	2	1	1	scara3	scara3	S	I	M	L	G	T	T	D	L	L	R	E	Cor
OL 1	97	2	1	1	outlier	outlier	S	S	L	I	P	P	H	N	A	K	D	G	N/A
C5 1 sra	93	2	2	1	sra	sra	C	R	S	L	G	Y	L	D	V	E	R	V	Cor
C6 2	22	3	1	1	scara3	scara3	A	G	Q	L	G	P	E	V	R	K	L	Q	N/A
scara3 1, sra 1	92	3	1	1	sra	scara4	V	A	L	L	G	L	Y	I	L	M	F	G	N/A
C7 3	27	4	1	2	scara3	scara3	Q	A	T	L	G	A	S	S	Q	R	I	S	Cor
scara3 3	28	4	1	2	scara3	scara3	Q	A	T	L	G	V	S	S	Q	R	I	S	Cor
	31	4	1	2	scara3	scara3	Q	A	I	L	G	V	S	S	Q	R	I	S	Cor
C8 17	58	4	2	1	scara5	scara5	C	R	M	L	G	F	R	G	V	E	E	V	Cor
scara5 16	78	4	2	1	scara5	scara5	C	R	M	L	G	F	R	G	V	E	E	V	Cor
sra 1	85	4	2	1	sra	sra	C	R	S	L	G	Y	R	G	V	K	S	V	Misplaced
C9 13	79	7	2	1	sra	sra	C	R	S	L	G	Y	P	G	V	Q	A	V	Cor
sra 13	80	7	2	1	sra	sra	C	R	S	L	G	Y	P	G	V	Q	A	V	Cor
	95	7	2	1	sra	sra	C	R	S	L	G	Y	P	G	V	Q	A	V	Cor

Summary

Total entities: 98   3 OLs were implanted, Cluster C6 undefined. They are not countec Total=95   Misplaced 4 out out of 93.

Entity Cluster Placement Accuracy: Since the only Cra E94 is in C6, it did not improve the placement. Accuracy still = (93-4)/93= 95.70%

(a)

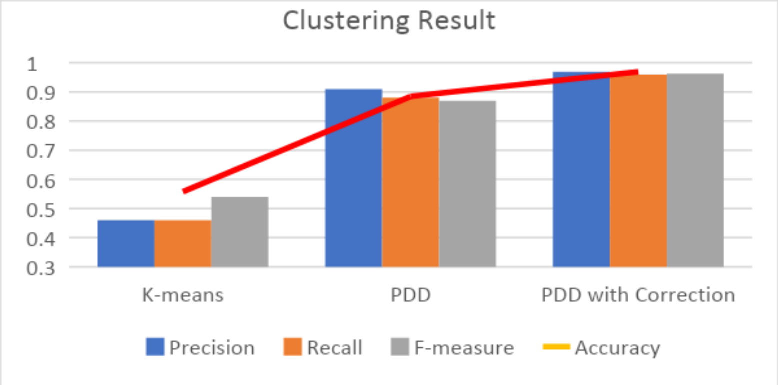

(b)

Supplementary Figure 4. Results of Unsupervised Learning on Class A Scavenger Receptor (SR-A) dataset. (a). The clustering results obtained by PDD concur with the results in the Knowledge Base (Supplementary Figure 3). The cluster in DSU[1 1 1] contains entities pertaining to *Marco, Scara5* and *Sra* as revealed also in the Knowledge Base. As for other clusters, each of them is associated with an implicit class. For example DSU[1 2 1] with *Scara4*, DSU[4 1 2] a small cluster of *Scara*3 with three samples and DSU[7 1 1] with *Sra* and even a single entity E93 of *Sra* in C5 separating from other clusters. Note that the OLs found in Knowledge Base were still placed into the correct clusters as indicated by their implicit class label showing the strength of PDD in Entity Clustering. Discounting C6 and 3 OLs, with 4 misplaced, we obtained a placement accuracy of (93- 4)/93 = 95.70% both before and after Cra was obtained. (**b).** We compare the PDD clustering results with that of K-Means. In all scores, we show that PDD outperforms K-Means significantly.

<table>
<tr><th>Seq ID \ APC Column Position</th><th>Class</th><th>1
234</th><th>2
235</th><th>3
236</th><th>4
237</th><th>5
238</th><th>6
239</th><th>7
240</th><th>8
241</th><th>9
242</th><th>10
243</th><th>11
244</th><th>12
245</th><th>Sequence Position</th></tr>
<tr><td>1 - 7, 10 -12, 19-20</td><td>marco</td><td>C</td><td>R</td><td>M</td><td>L</td><td>G</td><td>Y</td><td>S</td><td>K</td><td>G</td><td>R</td><td>A</td><td>L</td><td>455-463</td></tr>
<tr><td>8</td><td>marco</td><td>C</td><td>R</td><td>M</td><td>L</td><td>G</td><td>F</td><td>S</td><td>S</td><td>G</td><td>R</td><td>A</td><td>I</td><td>438</td></tr>
<tr><td>9</td><td>marco</td><td>C</td><td>R</td><td>M</td><td>L</td><td>G</td><td>Y</td><td>S</td><td>S</td><td>G</td><td>S</td><td>P</td><td>V</td><td>330</td></tr>
<tr><td>13</td><td>marco</td><td>C</td><td>R</td><td>M</td><td>L</td><td>G</td><td>Y</td><td>S</td><td>R</td><td>G</td><td>T</td><td>A</td><td>L</td><td>411</td></tr>
<tr><td>14</td><td>marco</td><td>C</td><td>R</td><td>M</td><td>L</td><td>G</td><td>Y</td><td>S</td><td>S</td><td>G</td><td>L</td><td>A</td><td>T</td><td>472</td></tr>
<tr><td>15</td><td>marco</td><td>C</td><td>R</td><td>M</td><td>L</td><td>G</td><td>Y</td><td>S</td><td>R</td><td>G</td><td>D</td><td>G</td><td>Y</td><td>408</td></tr>
<tr><td>18</td><td>marco</td><td>C</td><td>R</td><td>M</td><td>L</td><td>G</td><td>Y</td><td>S</td><td>R</td><td>A</td><td>V</td><td>Q</td><td>A</td><td>212</td></tr>
<tr><td>21</td><td>marco</td><td>C</td><td>R</td><td>M</td><td>L</td><td>G</td><td>Y</td><td>S</td><td>S</td><td>G</td><td>K</td><td>G</td><td>F</td><td>424</td></tr>
<tr><td>22, 23</td><td>scara3</td><td>S</td><td>I</td><td>M</td><td>L</td><td>G</td><td>T</td><td>T</td><td>D</td><td>L</td><td>L</td><td>R</td><td>E</td><td>403-404</td></tr>
<tr><td>25 - 28, 31-32 , 41 -42</td><td>scara3</td><td>S</td><td>L</td><td>M</td><td>L</td><td>G</td><td>T</td><td>T</td><td>D</td><td>L</td><td>L</td><td>R</td><td>E</td><td>396-404</td></tr>
<tr><td>24</td><td>scara3</td><td>A</td><td>G</td><td>Q</td><td>L</td><td>G</td><td>P</td><td>E</td><td>V</td><td>R</td><td>K</td><td>L</td><td>Q</td><td>121</td></tr>
<tr><td>29, 30, 33</td><td>scara3</td><td>Q</td><td>A</td><td>T</td><td>L</td><td>G</td><td>A | V</td><td>S</td><td>S</td><td>Q</td><td>R</td><td>I</td><td>S</td><td>279-280</td></tr>
<tr><td>43, 46-50, 52, 53, 55, 56, 59-61,63,67</td><td>scara4</td><td>V</td><td>A</td><td>I</td><td>L</td><td>G</td><td>Y</td><td>K</td><td>V</td><td>V</td><td>E</td><td>K</td><td>M</td><td>50-55</td></tr>
<tr><td>44, 64</td><td>scara4</td><td>V</td><td>A</td><td>I</td><td>L</td><td>G</td><td>Y</td><td>K</td><td>V</td><td>V</td><td>E</td><td>K</td><td>M</td><td>100, 112</td></tr>
<tr><td>45, 51, 54, 57</td><td>scara4</td><td>V</td><td>A</td><td>I</td><td>L</td><td>G</td><td>Y</td><td>K</td><td>V</td><td>V</td><td>E</td><td>K</td><td>M</td><td>35-54</td></tr>
<tr><td>58</td><td>scara4</td><td>V</td><td>A</td><td>I</td><td>L</td><td>G</td><td>Y</td><td>K</td><td>V</td><td>V</td><td>E</td><td>K</td><td>M</td><td>81</td></tr>
<tr><td>62</td><td>scara4</td><td>V</td><td>A</td><td>V</td><td>L</td><td>G</td><td>Y</td><td>K</td><td>V</td><td>V</td><td>Q</td><td>R</td><td>V</td><td>71</td></tr>
<tr><td>65</td><td>scara4</td><td>V</td><td>A</td><td>I</td><td>L</td><td>G</td><td>Y</td><td>K</td><td>V</td><td>V</td><td>E</td><td>K</td><td>M</td><td>54</td></tr>
<tr><td>66</td><td>scara4</td><td>V</td><td>A</td><td>V</td><td>L</td><td>G</td><td>Y</td><td>K</td><td>V</td><td>V</td><td>Q</td><td>R</td><td>V</td><td>57</td></tr>
<tr><td>68. 69, 71-83, 85, 88, 89</td><td>scara5</td><td>C</td><td>R</td><td>M</td><td>L</td><td>G</td><td>F</td><td>R | H | P</td><td>G</td><td>V | A</td><td>E | K</td><td>E | D</td><td>V</td><td>430-435</td></tr>
<tr><td>70</td><td>scara5</td><td>C</td><td>R</td><td>M</td><td>L</td><td>G</td><td>F</td><td>R</td><td>G</td><td>V</td><td>E</td><td>E</td><td>V</td><td>393</td></tr>
<tr><td>86</td><td>scara5</td><td>C</td><td>R</td><td>M</td><td>L</td><td>G</td><td>Y</td><td>R</td><td>G</td><td>A</td><td>T</td><td>E</td><td>V</td><td>347</td></tr>
<tr><td>90 - 102, 104 - 106</td><td>sra</td><td>C</td><td>R</td><td>S</td><td>L</td><td>G</td><td>Y</td><td>P | R | Q</td><td>G</td><td>V</td><td>Q|L|R|K</td><td>A</td><td>V</td><td>374-390</td></tr>
<tr><td>103</td><td>sra</td><td>V</td><td>A</td><td>L</td><td>L</td><td>G</td><td>L</td><td>Y</td><td>I</td><td>L</td><td>M</td><td>F</td><td>G</td><td>52</td></tr>
</table>

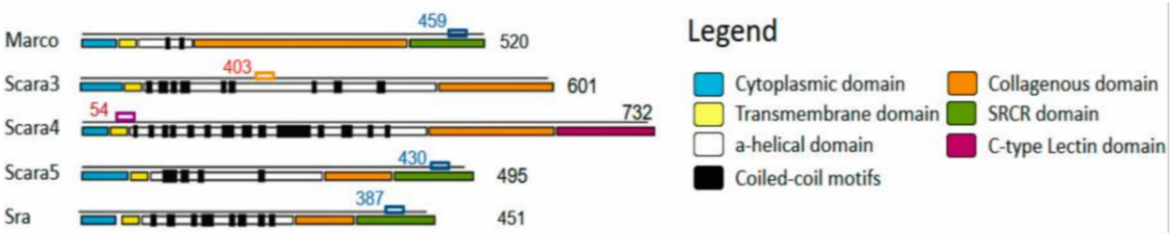

Supplementary Figure 5. Discovering and locating patterns associated with Functional Domains. Column 1 shows the position of the sequences of the aligned patterns found by PDD. The last column indicates their sequence position in the functional domains of the SR-A families as shown in the bottom figure. PDD discovered and located patterns in the functional domains of the family.

Discussion: Interpretability and traceability for Scientific Exploration

Because of the AV-association ground truth knowledge provided in this dataset [2], we will use it to demonstrate the interpretability and traceability of PDD. Supplementary Figure 5 shows the capability of PDD in discovering and locating patterns associated with functional domains scattered in the sequences of the SR-A family (legend in Supplementary Figure 5). It displays the sequence position in the five functional domains of the SR-A family as shown in the last column confirmed by existing literatures [2]. It is intriguing to note that from the disentangled patterns revealed in Supplementary Figures 3(a)(c)(d), the location of the *Scara3* and *Scara4* group (in

red numeral fonts) and the *Marco, Scara5* and *Sra* group (in blue numeral fonts) are sharing more common patterns as they reside on same location in the SR-A family as shown in the bottom diagram. Linking all this information in the Knowledge Bases and Entity Clusters together with class association and pattern locations, PDD renders a comprehensive analysis and an explainable and verifiable Knowledge Base in support of further scientific/clinical exploration

Case Study 3: Breast Cancer

Data and Problems

Detection of tumors at the earliest possible stage is of paramount importance for cancer treatment, and a missed diagnosis may lose critical time that the patient needs. In the past, most ML researchers used this data for testing and evaluating supervised and unsupervised classification. This paper exemplifies how PDD can obtain correct pattern-class associations and identify the missed diagnosis and the misdiagnosis in the borderline cases from this large dataset.

Cancer Wisconsin dataset [5] is a health care benchmark dataset taken from UCI repository, which is a well-studied classical dataset with 699 cases for discriminating the instances of two possible classes: Benign 458 cases (distribution=65.5%) and Malignant (distribution=34.5%).

PDD Knowledge Base

Since the Breast Cancer dataset consists of 699 cases, we just present the Summarized Knowledge Base for the dataset wCL integrated with the results obtained from nCL. In this Knowledge Base, we only display several representatives of the correctly classified entities, but all the anomalies identified and rectified to explain how they were handled.

1) **All-in-One Knowledge Base.** Supplementary Figure 6 displays the Summarized Knowledge Base obtained from wCL. It shows succinct relations among the Knowledge Space, the Pattern Space (the top figure) and the Entity Space as well as the integrated class association results

obtained from wCL and nCL at the bottom of the Entity Space (the bottom figure). It gives a unified view of the high-level results in a traceable manner by linking information/knowledge from input data, individual entities, pattern sub-groups and Entity Clusters (Supplementary Figure 7). It provides class association with inconsistency check of the class status obtained and discrepancy rectified for further examination. It also relates the result obtained from the Knowledge Base and Entity Clusters to render a full picture using supervised information within an unsupervised process.

2) **AV-Association Disentanglement and Pattern-Class Association with Anomaly Correction.** The Knowledge Space (Top figure of Supplementary Figure 6) shows that PDD obtained almost perfect disentanglement. We found the DSUs for Benign and Cancerous are on the opposite side of the PCs as indicated by the second index of their DSU code "1" or "2" (DSU[1 1 1] and DSU[1 2 1] in DS1).

In the Pattern Space, we noticed that AVs making up of the patterns for two classes are distinct, indicating the proficient use of the indicants in the dataset and the effectiveness of the discretization scheme. We also found that all patterns seldom have the "either this or that AV" case, like those common in other data mining and pattern discovery models, except one case in DSU[1 1 1] where the AVs of the either-or case are adjacent intervals. We also observed that disentangled patterns, in the bottom rows, not containing class label as an AV (with no label on the class label column) are subsets of the unions of patterns of those with class label and occurring on entities with the same implicit class label. This indicates that PDD can detect AV-associations associated with classes as primary sources with and without the class label given.

Summarized PDDKB wCL With class labels in the dataset

Knowledge Space						Pattern Space									
DS* / DSU			Primary Source / Pattern Occurrecnce		Statistics	Patterns / AVs									
DS	AVG	AVSG	Benign	Cancerous	Order	Class	Clump Thickness	Cell size Uniformity	Cell Sahpe Unifromtity	Marginal Adhesion	Single Ep. Cell Size	Bare Nuclei	Bland Chromatin	Normal Nucleoli	Mitosis
1	1	1	4305		4_9	Benign	[1 3)/[3 5)	[1 3)	[1 3)	[1 3)	[2 3)	[1 3)	[1 2)/[2 3)	[1 2)	1
1	1	2	164		2	Benign	[3 5)								
1	1	3	358	5	2*	Benign	[1 3)	[1 3)	[1 3)	[1 3)	[1 2)	[1 3)	[2 3)	[1 2)	1
1	2	1		110	9	Cancerous	[5 10)	[3 10)	[3 10)	[3 10)	[3 10)	[3 10)	[3 10)	[2 10)	
1	2	2		86	9*	Cancerous	[5 10)	[3 10)	[3 10)	[3 10)	[3 10)	[3 10)	[3 10)	[2 10)	2
1	2	3		43	9*	Cancerous	[5 10)	[3 10)	[3 10)	[3 10)	[3 10)	[3 10)	[3 10)	[2 10)	7
1	2	4		111	9*	Cancerous	[5 10)	[3 10)	[3 10)	[3 10)	[3 10)	[3 10)	[3 10)	[2 10)	3
1	2	5		5	8	Cancerous		[3 10)	[3 10)	[3 10)	[3 10)	[3 10)		[2 10)	8
1	2	6		122	8 *	Cancerous	[5 10)	[3 10)	[3 10)	[3 10)	[3 10)	[3 10)	[3 10)	[2 10)	4
1	2	7		209	5_8	Cancerous	[5 10)	[3 10)	[3 10)	[3 10)	[3 10)	[3 10)	[3 10)	[2 10)	10
1	2	8		3	6	Cancerous		[3 10)	[3 10)		[3 10)	[3 10)			6
2	1	1	355		4	Benign		[1 3)	[1 3)			[1 3)			
2	2	1		201	4	Cancerous		[3 10)	[3 10)			[3 10)			
3	1	1	316	2	3			[1 3)	[1 3)		[2 3)				
3	2	1	16	204	3			[3 10)	[3 10)		[3 10)				
4	1	1	7	149	5		[5 10)	[3 10)	[3 10)			[3 10)		[2 10)	
4	2	1	324		4			[1 3)	[1 3)			[1 3)		[1 2)	
5	1	1	278		4				[1 3)		[2 3)	[1 3)		[1 2)	
5	2	1	9	162	4				[3 10)		[3 10)	[3 10)		[2 10)	

Knowledge Space			Entity Space																																		
DS* / DSU			Explicit Class Labels / EIDs																																		
DS	AVG	AVSG	1	2	4	28	37	113	130	142	146	161	173	175	194	212	218	257	395	427	458	459	487	504	522	554	579	582	606	616	617	624	642	659	675	678	699
1	1	1	7																		20																
1	1	2											1	1	1																						
1	1	3																								5											
1	2	1																				1															
1	2	2																																			
1	2	3																																			
1	2	4																																			
1	2	5																																			
1	2	6																																			
1	2	7																																			
1	2	8																																			
2	1	1	1																		1																
2	2	1																				1		1	1		1	1				1		1		1	1
3	1	1	1																		1								1				1				
3	2	1		1	1	1		1	1	1	1	1	1	1	1	1		1	1	1		1			1		1	1		1		1		1	1	1	1
4	1	1		1	1			1		1	1	1						1				1															
5	2	1	1																		1																
5	1	1	1																		1																
5	2	1		1	1			1		1	1	1		1	1			1				1									1						1
Row A: Class Satus (wCL)			1	2	4	28	37	113	130	142	146	161	173	175	194	212	218	257	395	427	458	459	487	504	522	554	579	582	606	616	617	624	642	659	675	678	699
Row B: Class Status (nCL)			1	2	4	28	37	113	130	142	146	161	173	175	194	212	218	257	395	427	458	459	487	504	522	554	579	582	606	616	617	624	642	659	675	678	699
Row C: Class Status (final)			1	2	4	28	37	113	130	142	146	161	173	175	194	212	218	257	395	427	458	459	487	504	522	554	579	582	606	616	617	624	642	659	675	678	699

Benign Cancerous OL Und Inc Cra(B -> C) Cra(C->B)

Final
Correct (Cor): #Benign=444; #Cancerous=238
Class Readjusted (Cra): #(Benign->Cancerous)=13; #(Cancerous->Benign)=3
Undetermined (Und) = 1

Accuracy
Before Readjusted (Cra): Mislabels=16; Outliers=12; Undetermined=1; Incorrect=2; Accuracy = (699-16-12-3)/699 = 95/57%
After Readjusted (Cra): Incorrect = 2; Undetermined = 1; Accuracy = (699-2-1)/699=99.57%

Supplementary Figure 6. Knowledge Base for Breast Cancer Dataset with Class Label given. This is the Summarized Knowledge Base where the class label is used as a normal attribute. In this figure the class color code for Benign and Cancerous classes are red and green respectively. In each of the 5 DS in the Knowledge Space, Benign and the Cancerous classes are on the opposite side of the PC as indicated by the second index ("1" or "2") in the DSU. The primary sources show superb disentanglement. In the Pattern Space, note that the set of AVs making up the patterns for two classes are distinct but with minor variation in the subgroup (AV-Subgroups). Note that the patterns with class labels are quite similar to those without. In the Entity Space, we show only a few representative entities of Benign, Cancerous and Outliers in light green, red and grey shade respectively. The remaining ones were anomalies with readjusted class labels (denoted by Cra). The third row on the top section of the table shows the EIDs with a given class label in class color-code. In the bottom section, Row A denotes the class status found from wCL and Row B from nCL. Row C shows the result after the integration of wCL and nCL according to the class association rules. Note that a few of Cra were found in wCL. We replaced the class status Inc found from nCl to Cra. Since all the Cra were absent before, they were considered as mislabeled. Thus, with 12 OLs, 4 Und and 16 mislabeled, the class association accuracy before Cra is (699-12-3-16)/699=95.57% Since after Cra and class status integration, only a single Und was retained. We attained an accuracy of 696/699=99.57%, comparable with the best results for supervised ML.

In the Entity Space, we displayed only a few representative successful classified cases: E1 and E458 among the 438 Benign and E459 and E699 among the 237 Cancerous cases. The remaining entities were found to be anomalies either in wCL or nCL. For the wCL cases, the anomalies (outliers or mislabels) can be found from the pattern possessed by the entities as indicated in the EID column in the figure. For example, E2 with implicit class label Benign was found by PDD as containing only Cancerous pattern from DSU[3 2 1] and DSU[5 2 1], but no Benign pattern. Hence, it was considered as a mislabel and its class label was readjusted as Cancerous (in darker red color, row A). It was confirmed later in entity clustering (Supplementary Figure 7) by the Cancerous patterns (in AVs with dark red shade) it possesses. Since this was confirmed in wCL as Cra, the misclassified status Inc (in blue color-code, row B) in nCL was dismissed, coming up with the class label of Cancerous as its final class status (row C). In E37, PDD found no significant pattern in its EID column in wCL and thus considered it as an outlier (OL) (row A). However, since other information was obtained in nCL (row B) (not shown here) in the DSU associated with Benign, PDD retained its implicit class status as a Benign. Based on the class association rules given in the Methodology, the final class status was assigned to each entity in row C. We list the rules again here as a direct reference to the readers.

The Entity Class-Association Rules

1. An entity is assigned as a Cor, an Inc or a Und when found from wCL, but its class label will be readjusted to Cra accordingly if found and confirmed in wCL/nCL. (Rationale: we give strongest weight to the ground truth unless label discrepancy is spotted).
2. An entity is an OL or an Und only if affirmed in both wCL and nCL but assumes the class status of the class label found in either.

Note that E1, E175, E194, E458, E459, E504, E617, E624 and E699 were considered as Cor by rule 1 since none of them is Cra. E37 and E487 were OLs found in wCL, but additional information in nCL showed that they were Benign and Cancerous respectively (Row B). Therefore, the final class label was assigned to them as Benign and Cancerous correspondingly (Row C).

With these rules, we obtained the integrated class association results as displayed at the bottom of the Entity Space. The accuracy rate in entity class association was 95.57% before Cra and 99.57% after. We will have a detailed discussion in the interpretability and traceability section after the entity clustering section.

Cluster ID	EID	DS	AVG	AVSG	Implicit	Cra	Clump Thickness	Cell Size Uniformity	Cell Shape Uniformity	Marginal Adhesion	Single Epithelial Cell Size	Bare Nuclei	Bland Chromatin	Normal Nucleoli	Mitosis	Placement
C1 384	1	1	1	1	Benign	Benign	[5 10)	[1 3)	[1 3)	[1 3)	[2 3)	[1 3)	[3 10)	[1 2)	1	Cor
B = 383	3	1	1	1	Benign	Benign	[3 5)	[1 3)	[1 3)	[1 3)	[2 3)	[1 3)	[3 10)	[1 2)	1	Cor
Cra = 1	5	1	1	1	Benign	Benign	[3 5)	[1 3)	[1 3)	[3 10)	[2 3)	[1 3)	[3 10)	[1 2)	1	Cor
	458	1	1	1	Benign	Benign	[1 3)	[1 3)	[1 3)	[1 3)	[3 10)	[1 3)	[1 2)	[1 2)	1	Cor
	642	1	1	1	Cancerous	Benign	[5 10)	[1 3)	[1 3)	[1 3)	[3 10)	[3 10)	[1 2)	[1 2)	2	Cra, Cor
C2 144	10	1	1	3	Benign	Benign	[1 3)	[1 3)	[1 3)	[1 3)	[3 10)	[1 3)	[3 10)	[1 2)	1	Cor
B = 143	18	1	1	3	Benign	Benign	[3 5)	[1 3)	[1 3)	[1 3)	[3 10)	[1 3)	[2 3)	[1 2)	1	Cor
Cra = 1	22	1	1	3	Benign	Benign	[3 5)	[1 3)	[1 3)	[1 3)	[3 10)	[1 3)	[2 3)	[1 2)	1	Cor
	398	1	1	3	Benign	Benign	[5 10)	[1 3)	[1 3)	[1 3)	[3 10)	[1 3)	[2 3)	[1 2)	1	Cor
	554	1	1	3	Cancerous	Benign	[3 5)	[1 3)	[1 3)	[3 10)	[3 10)	[3 10)	[2 3)	[1 2)	1	Cra, Cor
C3 159	2	1	2	1	Benign	Cancerous	[5 10)	[3 10)	[3 10)	[3 10)	[3 10)	[3 10)	[3 10)	[2 10)	1	Cra, COr
B = 4	4	1	2	1	Benign	Cancerous	[5 10)	[3 10)	[3 10)	[1 3)	[3 10)	[3 10)	[3 10)	[2 10)	1	Cra, Cor
C = 143	28	1	2	1	Benign	Cancerous	[5 10)	[3 10)	[3 10)	[3 10)	[3 10)	[0 1)	[3 10)	[2 10)	1	Cra, Cor
Cra: B->C=11	130	1	2	1	Benign	Cancerous	[5 10)	[3 10)	[3 10)	[3 10)	[3 10)	[1 3)	[3 10)	[2 10)	1	Cra, Cor
Cra: C->B=1	146	1	2	1	Benign	Cancerous	[5 10)	[3 10)	[3 10)	[1 3)	[3 10)	[3 10)	[3 10)	[2 10)	1	Cra, Cor
	161	1	2	1	Benign	Cancerous	[5 10)	[3 10)	[3 10)	[3 10)	[3 10)	[3 10)	[3 10)	[2 10)	1	Cra, Cor
	173	1	2	1	Benign	Benign	[3 5)	[3 10)	[3 10)	[3 10)	[3 10)	[0 1)	[3 10)	[2 10)	1	Cor
	175	1	2	1	Benign	Cancerous	[3 5)	[3 10)	[3 10)	[3 10)	[3 10)	[3 10)	[3 10)	[2 10)	1	Inc. Cor
	194	1	2	1	Benign	Cancerous	[3 5)	[3 10)	[3 10)	[3 10)	[3 10)	[3 10)	[3 10)	[2 10)	1	Inc, Cor
	212	1	2	1	Benign	Cancerous	[5 10)	[3 10)	[3 10)	[1 3)	[3 10)	[1 3)	[3 10)	[1 2)	1	Cra, COr
	218	1	2	1	Benign	Cancerous	[5 10)	[3 10)	[3 10)	[1 3)	[3 10)	[1 3)	[3 10)	[1 2)	1	Cra, Cor
	242	1	2	1	Benign	Benign	[3 5)	[3 10)	[1 3)	[3 10)	[3 10)	[3 10)	[3 10)	[2 10)	1	Cor
	257	1	2	1	Benign	Cancerous	[5 10)	[3 10)	[3 10)	[3 10)	[3 10)	[3 10)	[3 10)	[2 10)	1	Cra, Cor
	395	1	2	1	Benign	Cancerous	[5 10)	[3 10)	[3 10)	[3 10)	[3 10)	[1 3)	[3 10)	[1 2)	1	Cra, Cor
	427	1	2	1	Benign	Cancerous	[5 10)	[3 10)	[3 10)	[1 3)	[3 10)	[1 3)	[3 10)	[2 10)	1	Cra, Cor
	459	1	2	1	Cancerous	Cancerous	[5 10)	[3 10)	[3 10)	[3 10)	[3 10)	[3 10)	[3 10)	[2 10)	1	Cor
	606	1	2	1	Cancerous	Benign	[5 10)	[1 3)	[1 3)	[1 3)	[3 10)	[3 10)	[3 10)	[2 10)	1	Cra, Misplaced
	699	1	2	1	Cancerous	Cancerous	[3 5)	[3 10)	[3 10)	[3 10)	[3 10)	[3 10)	[3 10)	[2 10)	1	Cor
C4 = 96	113	1	2	2	Benign	Cancerous	[5 10)	[3 10)	[3 10)	[3 10)	[3 10)	[3 10)	[3 10)	[2 10)	2	Cra, Cor
B = 0	463	1	2	2	Cancerous	Cancerous	[5 10)	[3 10)	[3 10)	[3 10)	[3 10)	[3 10)	[3 10)	[1 2)	2	Cor
C=26	472	1	2	2	Cancerous	Cancerous	[5 10)	[3 10)	[3 10)	[1 3)	[3 10)	[3 10)	[3 10)	[2 10)	2	Cor
Cra:B->C=1	477	1	2	2	Cancerous	Cancerous	[5 10)	[3 10)	[3 10)	[1 3)	[3 10)	[3 10)	[3 10)	[2 10)	2	Cor
	697	1	2	2	Cancerous	Cancerous	[5 10)	[3 10)	[3 10)	[3 10)	[3 10)	[3 10)	[3 10)	[2 10)	2	Cor
C5 8	481	1	2	3	Cancerous	Cancerous	[5 10)	[3 10)	[3 10)	[3 10)	[3 10)	[3 10)	[3 10)	[2 10)	7	Cor
B=0	497	1	2	3	Cancerous	Cancerous	[3 5)	[3 10)	[3 10)	[3 10)	[3 10)	[3 10)	[3 10)	[2 10)	7	Cor
C=8	507	1	2	3	Cancerous	Cancerous	[5 10)	[3 10)	[3 10)	[3 10)	[3 10)	[3 10)	[3 10)	[2 10)	7	Cor
	694	1	2	3	Cancerous	Cancerous	[5 10)	[3 10)	[3 10)	[3 10)	[3 10)	[3 10)	[3 10)	[2 10)	7	Cor
C6 32	142	1	2	4	Benign	Cancerous	[5 10)	[3 10)	[3 10)	[3 10)	[3 10)	[3 10)	[3 10)	[2 10)	3	Cra, Cor
B=0	468	1	2	4	Cancerous	Cancerous	[5 10)	[3 10)	[3 10)	[3 10)	[3 10)	[3 10)	[3 10)	[2 10)	3	Cor
C=31	473	1	2	4	Cancerous	Cancerous	[5 10)	[3 10)		[3 10)	[3 10)	[3 10)	[3 10)	[2 10)	3	Cor
Cra:B->C=1	496	1	2	4	Cancerous	Cancerous	[5 10)	[3 10)	[3 10)	[1 3)	[3 10)	[3 10)	[3 10)	[2 10)	3	Cor
	695	1	2	4	Cancerous	Cancerous	[5 10)	[3 10)	[3 10)	[3 10)	[3 10)	[3 10)	[3 10)	[2 10)	3	Cor
C7 3	504	1	2	5	Cancerous	Cancerous	[1 3)	[3 10)	[3 10)	[3 10)	[3 10)	[3 10)	[2 3)	[2 10)	1	Cor
B=0	514	1	2	5	Cancerous	Cancerous	[3 5)	[3 10)	[3 10)	[3 10)	[3 10)	[3 10)	[3 10)	[2 10)	8	Cor
C=3	630	1	2	5	Cancerous	Cancerous	[3 5)	[3 10)	[3 10)	[3 10)	[3 10)	[3 10)	[3 10)	[2 10)	8	Cor
C8 12	461	1	2	6	Cancerous	Cancerous	[5 10)	[3 10)	[3 10)	[3 10)	[3 10)	[3 10)	[3 10)	[2 10)	4	Cor
B=0	464	1	2	6	Cancerous	Cancerous	[5 10)	[3 10)	[1 3)	[3 10)	[3 10)	[3 10)	[3 10)	[2 10)	4	Cor
C=12	485	1	2	6	Cancerous	Cancerous	[5 10)	[1 3)	[3 10)	[1 3)	[3 10)	[1 3)	[3 10)	[2 10)	4	Cor
	627	1	2	6	Cancerous	Cancerous	[5 10)	[3 10)	[3 10)	[3 10)	[3 10)	[3 10)	[3 10)	[2 10)	4	Cor
C9 14	491	1	2	7	Cancerous	Cancerous	[5 10)	[3 10)	[1 3)	[1 3)	[3 10)	[1 3)	[3 10)	[2 10)	10	Cor
B=0	494	1	2	7	Cancerous	Cancerous	[5 10)	[3 10)	[1 3)	[3 10)	[3 10)	[1 3)	[3 10)	[2 10)	10	Cor
C=14	498	1	2	7	Cancerous	Cancerous	[5 10)	[3 10)	[3 10)	[1 3)	[3 10)	[3 10)	[3 10)	[2 10)	10	Cor
	689	1	2	7	Cancerous	Cancerous	[5 10)	[3 10)	[3 10)	[3 10)	[3 10)	[1 3)	[3 10)	[2 10)	10	Cor
C10 3	85	2	1	1	Benign	Benign	[3 5)	[1 3)	[1 3)	[3 10)	[3 10)	[1 3)	[3 10)	[2 10)	1	Cor
B = 3	244	2	1	1	Benign	Benign	[5 10)	[1 3)	[1 3)	[1 3)	[3 10)	[1 3)	[3 10)	[2 10)	2	Cor
	254	2	1	1	Benign	Benign	[5 10)	[1 3)	[1 3)	[3 10)	[3 10)	[1 3)	[3 10)	[2 10)	1	Cor
C11 1 C=1	478	2	2	1	Cancerous	Cancerous	[5 10)	[3 10)	[3 10)	[1 3)	[3 10)	[3 10)	[2 3)	[1 2)	5	Cor
C12 2	62	4	1	1	Benign	Benign	[5 10)	[3 10)	[3 10)	[1 3)	[3 10)	[1 3)	[3 10)	[2 10)	1	Cor
B=2	162	4	1	1	Benign	Benign	[5 10)	[3 10)	[3 10)	[1 3)	[3 10)	[0 1)	[2 3)	[2 10)	1	Cor

Summary Total 699

Entity Cluster Placement: Cor = 679 Cra, Cor = 15 Cra, Misplaced = Mispalced = 4 Total Cor = 679+15=694

Accuracy of Entity Placem Before Cra: Before Cra (Accuracy is based on mispalcement of eneities not compying ot their impl: Accuracy = (699-19)/699 = 97.29%

Accuracy of Entity Placement after Cra: : After Cra with 3 misplacement: Accuacy = (699-3)/699 = 99.57%

(a)

Cluster #	C1	C2	C3	C4	C5	C6	C7	C8	C9	C10	C11	C12
**Class labels**	**B**	**B**	**C**	**C**	**C**	**C**	**C**	**C**	**C**	**B**	**C**	**B**
**Cluster size**	384	54	159	27	8	32	3	12	14	3	1	2
**PDD results (majority with distinct CL)**	378	52	139	24	8	31	3	11	14	3	1	2

(b)

Supplementary Figure. 7 Entity Cluster on Wisconsin Cancer Dataset. a. The clustering results with no class label given in the dataset is displayed. Column 1 displays the class associated with the clusters based on the implicit class labels of its majority entities and the sample size of subgroups therein. For example, in cluster C1, it contains 384 entities with 378 in the subgroup Benign (B), 6 outliers (OL) and 1 Cra which was labelled as cancerous but found possessing only Benign pattern(s). Column 2 shows its EIDs in the color-code of the final class status assigned in the Knowledge Base (Supplementary Figure 6). Columns 3 to 5 are the triple code of the DSU on which the entity in the cluster was found. Column 5 and Column 6 display the implicit class label and the final discovered/rectified class labels. The last column shows the entity cluster placement status — "Cor" denote correctly placed, "Misplaced" as incorrectly placed. The Summary at the bottom of the figure shows the efficacious unsupervised results. The entity placement accuracy 97.29% before Cra was estimated using the implicit class label of the entities being placed into the wrong clusters after PDD disentanglement and pattern discovery. That after Cra of 99.57% was estimated based on placement of the entities with the final class status. **b.** We give a summary of teh cluster size against the number of its majority members paertaing to a distinct class/group. It shows that PDD can discover clusters with different sizes correctly based on the statistical strength of their patterns in an unsupervised setting.

Entity Clustering Results

In this case study with cancer cytopathological data, the entity clustering results render overwhelming evidence of the efficacy of PDD's unsupervised approach. Based upon AV-association disentanglement, it unveiled superior Entity Cluster placement, i.e., placing entities into the right clusters (Supplementary Figure 7) while displaying details of each entity and clusters with statistically significant patterns to render transparency and reliability in support of the discovery.

In Supplementary Figure 7(a), we displayed some representatives of the correctly placed entities, and all anomalies found and Cra rectified together with their implicit and discovered class label. For entity placement assessment, we used the final class status results from the Knowledge Base in the discovered pattern column. Here we give a brief description of the entity clustering results rectified by the class-association results from the Knowledge Base (Supplementary Figure 7(a)). In the dataset, there are 699 cases with 458 pertaining to Benign (B) and 241 to Cancerous (C). In the first column, for each cluster, we displayed its cluster ID (#), the cluster size, the size of each

distinct group according to the final class status obtained from the Knowledge Base. For each cluster obtained, Supplementary Figure 7(b) summarized the cluster size against the number of its majority members pertaining to a distinct class/group.

From the Entity Clustering Results, we notice that:

a) PDD obtained clusters all of which consist of majority members pertaining to a distinct class, implying that the overall clustering result is correct.

b) The number of clusters was determined automatically based on the inherent disentangled patterns rather than the setting of optimization parameters.

c) The size of the clusters varied from 384 to one. The one contains only a single entity since it had no cluster to join, indicating PDD's capability to discover groups with imbalanced group size, even one with a single rare case.

d) Entity cluster placement accuracy before Cra was estimated by taking the implicit class label given to the entities as ground truth. It was found to be 97.29%. That after Cra was estimated as 99.28% where the final class status obtained from PDD after Cra was used instead. In Figure 5, we observe that PDD outperformed the existing models.

Discussion: Interpretability and Traceability for Scientific Exploration

PDD's unsupervised approach not influenced by prior knowledge or other physical/human factors while removing or rectifying confirmed errors or biases unnoticed in the data can unveil inherent and intrinsic information to provide succinct, transparent, reliable, interpretable, and traceable knowledge in the Knowledge Base obtained from wCL and nCL as well as Entity Clusters. The results in this case study and others fully demonstrate the high accuracy in class association and clustering as well as efficacious interpretability and traceability with statistical support and functional implication. As for result interpretation, for taxonomic data, taking classes as primary

sources is clear cut. However, for pathological and clinical data, to have precise labeling is a challenge due to variable factors such as the early stage of a disease/disorder, and existence of other factors/environments. Hence, we treat this case study as a statistical study within the scope of ML with such revealing capability of the borderline cases. Nevertheless, it unveils some intriguing results, suggesting cases of misdiagnosis, missed diagnosis and early diagnosis, a very important capability in cancer diagnosis and assessment as well as in clinical practice. It opens the door for further research.

Case Study 4: heart disease

Now we move on to a more variable dataset especially among the patients with the Presence and Absence of heart problems. It is a health care benchmark dataset from UCI repository [6] [7] containing 270 clinical records with 13 mixed-mode attributes in two possible classes: Absence or Presence (of heart disease), abbreviated as Abs and Prs. This represents a realistic interpretable clinical problem. We use it to illustrate the key capabilities of PDD and its special ways in dealing with anomalies and borderline cases.

PDD Knowledge Base

Figure 4 in Main presents the Knowledge Base obtained for the wCL with entity class association results integrated with those obtained from nCL. Again, in the Entity Space, we displayed only the anomalies and the representatives of the correctly classified cases. In this clinical data, we observed that three AVs in the Knowledge Base were not forming high-order patterns though they are traits related to heart problems. This implies that they do not have strong statistical interdependence among themselves or with other AVs and/or strong statistical association with the presence or absence of heart disease. In this Case Study, PDD did not use them and yet is able to obtain high

class association accuracy, we still retained them in the pattern space, linking to the entity space, to allow further reference for clinical judgement and treatment.

1) **AV-Association Disentanglement and Pattern-Class Association with Anomaly Identification and Correction.**

Patterns are high-order statistically significant associations. In clinical data, often, we find single individual traits which have association with disease classes but not much correlated with other factors. Such AVs do not form association patterns with other AVs in the data but are important by themselves for other clinical judgement in patients' care and treatment (like the 3 AVs (rpb, sc and fbs) (Figure 4 in Main). Figure 4 in Main shows strong evidence that DSUs and patterns associated with distinct primary sources/classes can be found in a clinical data set where the boundary between the disease and normal such as the "Absence" and "Presence" of heart problems is not as distinct as in the SR-A and the Breast Cancer cases. It also reveals borderline cases to alert further observation and judgement.

2) Knowledge Space of the Knowledge Base from wCL.

In the Knowledge Space of the summarized Knowledge Base obtained from wCL, we noticed superb AV-association disentanglement for the DSUs and the disentangled patterns, with class label or without class label (as unveiled in the class column in the Pattern Space). Those without class labels were associated with distinct primary sources/classes reflected by their pattern occurrences on entities with distinct implicit class label, substantiated by the success of entity class association as shown at the EID rows at the bottom section of the figure in the Entity Space. We observed that the AV-associations associated with the two classes were on the opposite side of the PC in the DS, containing distinct AVs on the same attributes in the disentangled patterns.

3) Pattern Space

In the Pattern Space, we observed the distinct patterns between these two groups. We found AVS rbp, sc and fbs are not forming high order patterns associated with classes since their possession by patients are not necessarily statistically interdependent. We also found low order association for patterns with no class label as an AV.

4) Entity Space

In the Entity Space of wCL, we observed strong pattern-to-class associations. We found 141 correct classifications out of 150 (94.00%) among Abs and 115 out of 120 (95.83%) among Prs. Like the results for the Wisconsin Breast Cancer case, we noticed that more subjects among the normal (Abs) were found having the disease (Prs) than the diseased person (Prs) found to be normal (Abs). In the Knowledge Base from wCl, we found 9 Abs possessing only Prs patterns while 2 Prs possess Abs patterns. In the final class association (Row C) we found 11 Abs possessing only Prs patterns and 5 Prs possessing only Abs patterns. The heart disease examples, together with those from the Breast Cancer data, show that PDD can bring in greater assurance and trust to attain early diagnosis and/or avoid missed diagnosis. It also renders more information for the borderline cases.

The entity class association results were summarized at the bottom of Figure 4 in Main. It displays all the patterns each entity possesses and how it can be used in the class label consistency check from the results of wCL in Row A and the results from nCL in Row B to obtain the integrated results in Row C. The class association accuracy from wCL before Cra came up as (141+115)/270 =94.81% and that from the integrated results turned out to be 100% after the Cra which were statistically affirmed. By plotting the union of the disentangled patterns onto the entities in entity clusters as we shall discuss later, we noticed that the rectified ones were mostly borderline cases as they contain both Abs and Prs patterns. When class labels (explicit or implicit) are given in the

data, they help both the classification as well as the discrepancies correction. Therefore, they may impact these cases to give higher class-association accuracy even up to 100% purely based on statistics. With transparency provided, such an accuracy can be further validated by tracing back to the patients' records or going through a closer examination. PDD brings in the alert — a step to assist clinical decision in general.

Entity Clustering

The heart disease data exemplifies the intriguing capability in relating entity clusters to the real world. Figure 4(b) in Main is the abridged results of Entity Clusters obtained without the influence of the class label. Each row is an entity with a distinct EDI. The columns in the table follow the convention of our previous entity clustering results. All those entities associated with a distinct DSU were found to belong to a distinct cluster pertaining to a class of its majority members. In PDD, it is the AV-association disentanglement that separates clusters. Hence, it does not require setting the number of clusters or finding optimal or fuzzy cluster configurations. The hierarchical clustering simply breaks a larger group into smaller groups based on the degree of overlapping of S-connected AVs/patterns. They share considerable similarity. The DSU triple code reveals clusters that are similar or distinct from each other. Clusters with the first two identical codes indicate that they are similar. If their second code is different, it implies that they are on the opposite side of the PC in a DS and thus distinct from each other. In PDD, entities forming a cluster are based on the patterns they possess. Their union pattern in its associated DSU reveals the characteristic and the underlying primary source of the cluster.

Column 3 of Figure 4(b) in Main listed the implicit (original) class label (in class-color code) of each entity and Column 4 that of its final discovered class status integrating the Cra results from both wCL and nCL. For E3, the implicit class label was Abs and found associated with Abs. It was

considered as correctly classified and thus correctly placed into the cluster (C1) associated with Abs. We denoted it as Cor in the entity placement column. E152 with a given implicit class label of Prs was found only possessing statistically significant patterns of Abs but none of the Prs pattern. Hence, its placement in an Abs cluster was considered as correct. E216 was labeled as Prs and was also found possessing patterns of Prs, but was placed into a Abs cluster. Therefore, it was considered misplaced. As for E260, it was labeled as an Prs but readjusted as an Abs while being placed into a Prs cluster. Hence, it was considered as misplaced.

To give a reasonable entity cluster placement accuracy, we used the class label assigned to the entities before or after Cra as the base. The placement is considered as Cor if the entity with its assigned class label is placed into a cluster pertaining to the class of the assigned class label. Based on this simple rule, we found from the full table of entity clustering results the number of entities with assigned class labels being placed into the wrong clusters. As Supplementary Figure 8(b) shows, we found 54 entities misplaced before Cra and 36 after. Hence, we have an accuracy of 80% before and 86.67% after Cra.

When we plotted the discovered patterns on the AV cells on the entities in the entity cluster with darker color-codes of the discovered class, we noticed that most of the misplaced were borderline cases in the sense that they possess significant patterns of both classes. This may explain why the entity clustering results were different from the class-association results. We shall address this notion in the discussion section.

Discussion: Pattern Transparency, Class Status Association and Entity clustering Results

In traditional ML, classification usually adopts a k-fold cross-validation process to get the average performance for assessing and selecting the best rules for the classifier through fine-tuning feature engineering and parameter selection. Since in the traditional ML models, there is no way to identify

and locate the anomalies and samples from uneven class distribution, the k-fold method is a good way to randomly distribute the samples and anomalies on the training and test sets to get the average performance. PDD discovers disentangled patterns disregarding where they are located in the data for class associations based on disentangled statistics, not relying on feature engineering or parameter tuning (in all the six case studies, we took the same set of parameters by default). Hence, from the theoretical view and experimental results of the six case studies, PDD, by and large, could identify and confirm the rectified anomalies, discover rare and imbalanced groups/classes, producing class-association rules to get high accuracy for class association in Knowledge Base and entity cluster placement disregards where they were placed in the dataset.

From Figure 4(a) in Main, we were surprised to find the 100% class-association accuracy after Cra whereas in the Entity Cluster, we got only 86.67% accuracy after Cra, a significant drop. One of the clues we noticed from the superimposed patterns is that most of the misplaced in the entity clusters are borderline cases. Since in the Knowledge Base, PDD class-association exploited the class label given in both wCL and nCL, the class label could play a determinant factor in those cases. For a problem when the borderline is fuzzy, the unsupervised method such as PDD based on disentangled patterns with transparency and statistical support may provide a less biased and interpretable approach for the clinicians to watch and go deeper, particularly for some more subtle cases.

The display in Knowledge Base and Entity Clusters show the importance of the transparency of the detected patterns and statistical evidence to justify the rectification of the label discrepancies in both the Knowledge Base and the entity clustering results — a unique capability of PDD that helps to interpret and improve the quality of class association and clustering. It will change our view of class-association and cluster evaluation as it offers a new way of anomalies adjustment

before the final acceptance of the results. While still providing a decision criterion, PDD will help clinical decision-making on anomalous cases and assist research and organization of the discovered knowledge which could be statistically and functionally confirmed.

Case Study 5: Thoracic Dataset – Imbalanced Class

To validate the capability of PDD for imbalance classification, another practically useful thoracic dataset was employed. The dataset described the surgical risk originally collected at Wroclaw Thoracic Surgery Centre for patients who underwent major lung resections for primary lung cancer in the years 2007-2011 [8]. It is composed of 470 samples with 14 attributes. To simulate the target scenario without requiring much tweaking, the numeric attributes PRE4, PRE5 and age were removed. The target attribute (taken as class label) is Risk. There are 400 samples labeled as *Risk=T* and only 70 samples labeled as *Risk=F*.

PDD Knowledge Base

Supplementary Figure 8 is the complete set of the Knowledge Base obtained from the Thoracic Dataset with imbalance classes. Supplementary Figures 8(a) and 8(b) show their Comprehensive Knowledge Base and Summarized Knowledge Base respectively, which entail the essential knowledge discovered by PDD and exemplify the efficacy of the all-in-one framework.

Like the previous experiments, the Knowledge Base consists of Knowledge Space, Pattern Space and Entity Space. Supplementary Figure 8(a) displays the Comprehensive Knowledge Base which contains a set of high-order patterns. Supplementary Figure 8(b) is the Summarized Knowledge Base with a union pattern in each DSU. When we examined whether PDD could discover the patterns associated with the minority class, we found that it discovered two AV-Groups in disentangled space, units DSU [1 1 1] and DSU [1 2 1] respectively. Each DSU is an AV-Subgroup containing interpretable patterns discovered. Supplementary Figure 8(b) is the Summarized

Knowledge Base displaying the union pattern of all the patterns in each DSU in the Comprehensive Knowledge Base (Supplementary Figure 8(a)). This shows that PDD can discover fewer patterns with specific associations to the classes to furnish easy interpretation. Furthermore, even with few patterns, PDD can reveal succinct and comprehensive characteristics (as exemplified in the synthetic case) of all given classes, even when the class distribution is imbalanced.

Comprehensive PDDKB																												
Knowledge Space							Pattern Space														Entity Space							
DSU			Primitive Source		Statistics		Patterns – Interdepedent AVs														F					T		
PC	AVG	AVSG	F	T	Residual	Order	Risk	Diagnosis	PRE6	PRE7	PRE8	PRE9	PRE10	PRE11	PRE14	PRE17	RE1	RE2	RE3	RE3	1	2	3	...	400	401	...	407
1	1	1	28	0	2.04	5	F				F			F	OC11				F					...			...	
1	1	1	28	0	1.56	5	F					F		F	OC11				F					...			...	
1	1	1	99	0	16.19	6	F		PRZ0		F	F	F	F								1		...	1		...	
1	1	1	28	0	11.75	7	F		PRZ0		F	F	F	F					F					...			...	
1	1	1	50	0	14.08	7	F		PRZ0		F	F	F	F	OC11									...			...	
1	1	1	14	0	9.94	8	F		PRZ0		F	F	F	F	OC11				F					...			...	
1	2	1	0	5	2.22	5	T		PRZ1				T	T					T					...		1	...	
1	2	1	0	4	7.38	5	T		PRZ2				T	T					T					...		1	...	
1	2	1	4	1	3.1	5	T				T		T	T					T					...		1	...	
1	2	1	0	3	2.98	6	T		PRZ1		T		T	T					T					...			...	

Note:
1. PC=Principal Component; AVG=Attribute Value Group; AVSG = Attribute Value Sub-Group;
2. F means Risk value is False; T means Risk value is True.

(a)

Sumamrized PDDKB																												
Knowledge Space							Pattern Space														Entity Space							
DSU			Primitive Source		Statistics		Patterns – Interdepedent AVs														F					T		
PC	AVG	AVSG	F	T	Residual	Order	Risk	Diagnosis	PRE6	PRE7	PRE8	PRE9	PRE10	PRE11	PRE14	PRE17	RE1	RE2	RE3	RE3	1	2	3	...	400	401	...	407
1	1	1	14	0	9.94	8	F		PRZ0		F	F	F	F	OC11				F			1				1		
1	2	1	0	3	2.98	6	T		PRZ1		T		T	T					T									

(b)

Supplementary Figure 8. Comprehensive and Summarized PDD Knowledge Base for Thoracic Dataset.

Entity Clustering

The Thoracic datasets exemplifies the predictive capability of PDD for imbalanced classes. The Thoracic dataset consists of 470 samples with 14 attributes, but only 70 patients were labeled as "risk" and 400 "no risk", showing quite an imbalanced dataset. Without any correction, for wCL, PDD obtained an association accuracy of 95.25%, biased toward the "no risk", and an accuracy of 68.57% for "risk", resulting in an average accuracy of 91.27% and a balanced accuracy of 82%.

This shows that without any anomaly detection, 429 entities were clustered in groups consistent with the original class label of their clustered entities. Here, we only show the clustering results of the remaining 41 entities (less than 10%) in Supplementary Figure 9, and find that, disregarding their implicit labelling, they were correctly placed into clusters with patterns pertaining to the other class as revealed by their patterns. Column 5 lists the implicit (original) class label of each entity and Column 6 that of its discovered class status obtained from the Knowledge Base. We highlight the patterns associated with Risk_F as green and the patterns associated with Risk_T as red. Supplementary Figure 9 clearly shows that the entities in the group DSU [1 1 1] and DSU [3 1 1] were covered by the patterns associated with Risk_F, but for some (listed in Supplementary Figure 9), they were labeled as Risk_T. Similarly, the entities in the group DSU [1 2 1] were covered by the patterns associated with Risk_T, but they were labeled as Risk_F. This result shows that PDD clustered these 41 cases according to their possessed patterns of a class not complying with that of the labeled class.

Case Study 6: Synthetic Dataset – Class Imbalance and Noise Tolerance

Problem and Data. In the Case Study 2, we have shown from Class A Scavenger Receptor (SR-A) APC dataset that PDD outperforms K-means in unsupervised clustering significantly in all scores based on the taxonomic ground truth (with 50% level by K-means vs 90% level by PDD) (Supplementary Figure 4(b)). Similarly, for the clustering results of the *Cytochrome C* APC dataset, PDD also outperforms K-means (Supplementary Figure 10). Now we would like to design a verifiable experiment to explore the noise tolerance capability of PDD in comparison with other ML Models. We did that in this case study by adding noise columns to the original clean and succinct Cytochrome C APC dataset.

Entity Clusters WCL

EID	DSU			Classes (Risk)		AVs											
	DS	AVG	AVSG	Implict	Disocvered	Diagnosis	PRE6	PRE7	PRE8	PRE9	PRE10	PRE11	PRE14	PRE17	PRE19	PRE25	PRE30	PRE32
405	1	1	1	T	F	DGN8	PRZ0	F	F	F	F	F	OC11	F	F	F	F	F
413	1	1	1	T	F	DGN3	PRZ0	F	F	F	F	F	OC13	F	F	F	T	F
423	1	1	1	T	F	DGN2	PRZ0	F	F	T	F	F	OC13	F	F	F	T	F
425	1	1	1	T	F	DGN4	PRZ0	F	F	F	F	F	OC12	F	F	F	T	F
427	1	1	1	T	F	DGN3	PRZ1	T	F	F	F	F	OC12	F	F	F	F	F
431	1	1	1	T	F	DGN5	PRZ0	F	F	T	F	F	OC12	F	F	F	F	F
432	1	1	1	T	F	DGN3	PRZ0	F	F	F	F	F	OC11	F	F	F	T	F
437	1	1	1	T	F	DGN3	PRZ0	F	F	F	F	F	OC12	T	F	F	T	F
445	1	1	1	T	F	DGN3	PRZ0	F	F	F	F	F	OC14	F	F	F	F	F
456	1	1	1	T	F	DGN3	PRZ0	F	F	F	F	F	OC11	F	F	F	T	F
459	1	1	1	T	F	DGN3	PRZ0	F	F	F	F	F	OC12	F	F	F	T	F
467	1	1	1	T	F	DGN2	PRZ1	F	F	F	T	F	OC14	T	F	F	F	F
403	3	1	1	T	F	DGN2	PRZ1	F	F	F	T	F	OC11	F	F	T	T	F
406	3	1	1	T	F	DGN3	PRZ1	F	F	F	T	T	OC11	F	F	F	T	F
408	3	1	1	T	F	DGN5	PRZ1	F	F	F	T	F	OC11	F	F	F	T	F
410	3	1	1	T	F	DGN5	PRZ0	F	F	F	T	F	OC12	F	F	F	T	F
429	3	1	1	T	F	DGN3	PRZ1	F	F	F	T	F	OC11	F	F	F	T	F
441	3	1	1	T	F	DGN5	PRZ1	F	F	F	F	T	OC11	F	F	F	T	F
444	3	1	1	T	F	DGN3	PRZ2	F	F	F	T	T	OC11	F	F	F	T	F
457	3	1	1	T	F	DGN2	PRZ1	F	F	F	F	T	OC11	F	F	F	T	F
463	3	1	1	T	F	DGN2	PRZ0	F	F	F	T	F	OC12	F	F	F	T	F
466	3	1	1	T	F	DGN5	PRZ1	F	F	F	T	F	OC11	F	F	F	T	F
74	1	2	1	F	T	DGN3	PRZ1	T	T	T	T	F	OC12	F	F	F	T	F
75	1	2	1	F	T	DGN4	PRZ1	F	T	F	T	F	OC12	F	F	F	T	F
118	1	2	1	F	T	DGN3	PRZ1	F	T	F	T	F	OC12	F	F	F	T	F
132	1	2	1	F	T	DGN3	PRZ1	F	T	F	T	T	OC12	F	F	T	T	F
136	1	2	1	F	T	DGN3	PRZ1	T	T	F	T	F	OC12	F	F	F	T	F
139	1	2	1	F	T	DGN3	PRZ1	F	T	T	T	F	OC12	F	F	F	T	F
145	1	2	1	F	T	DGN2	PRZ1	F	T	T	T	F	OC12	F	F	T	T	F
149	1	2	1	F	T	DGN3	PRZ1	F	T	F	T	T	OC12	F	F	F	T	F
179	1	2	1	F	T	DGN2	PRZ1	F	T	F	T	F	OC12	F	F	F	T	F
236	1	2	1	F	T	DGN2	PRZ1	F	T	F	T	F	OC12	F	F	F	T	F
248	1	2	1	F	T	DGN3	PRZ1	F	T	F	T	F	OC12	F	F	F	T	F
251	1	2	1	F	T	DGN3	PRZ1	F	T	F	T	T	OC12	F	F	F	T	F
253	1	2	1	F	T	DGN3	PRZ1	F	T	F	T	T	OC12	F	F	F	T	F
263	1	2	1	F	T	DGN2	PRZ1	F	T	F	T	F	OC12	F	F	F	T	F
277	1	2	1	F	T	DGN2	PRZ1	F	T	T	T	F	OC12	F	F	F	F	F
304	1	2	1	F	T	DGN3	PRZ1	F	T	F	T	F	OC12	F	F	F	T	F
330	1	2	1	F	T	DGN2	PRZ1	F	T	F	T	F	OC12	F	F	F	T	F
342	1	2	1	F	T	DGN3	PRZ1	F	T	F	T	F	OC12	F	F	F	T	F
366	1	2	1	F	T	DGN3	PRZ1	T	T	F	T	F	OC11	F	F	F	T	F

Supplementary Figure 9. Entity Clustering Results for Thoracic Dataset.

Entity Clustering Results and Discussion. As shown in Supplementary Figure 10(a) and 10(b), both K-means and PDD can obtain high clustering accuracy even without class information for the original APC1 dataset. After adding background noise to the APCs (with three to fifteen noise columns), the accuracy of K-means is significantly reduced whereas that of PDD remains essentially no change. This further validates the robustness of PDD against noise. Besides, K-means cannot render explicit displayable patterns/knowledge inherent in each cluster, whereas

PDD can. This further shows why PDD can reduce the effect of noise without feature engineering since it extracts statistically significant AV associations at even a deeper feature value level.

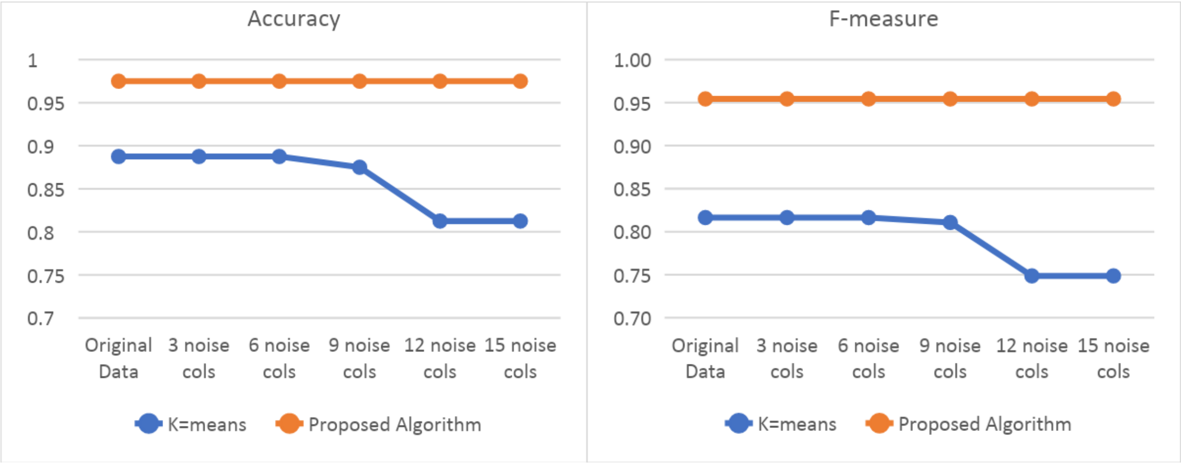

(a) **(b)**

Supplementary Figure 10. Results of Entity Clustering. **(a)** and **(b)**: Comparison of Segment Clustering Result on Cytochrome C APC Dataset with Noise Attributes (noise columns in **R**) added.

Reference

[1] P.-Y. Zhou, A. E. Lee, A. Sze-To and A. K. Wong, "Revealing Subtle Functional Subgroups in Class A Scavenger Receptors by Pattern Discovery and Disentanglement of Aligned Pattern Clusters," *Proteomes,* vol. 6, no. 1, p. 10, 2018.

[2] A. K. Wong and A. E. Lee, "Aligning and clustering patterns to reveal the protein functionality of sequences," *IEEE/ACM Transactions on Computational Biology and Bioinformatics (TCBB),* vol. 11, no. 3, pp. 548-560, 2014.

[3] F. J. Whelan, C. J. Meehan, G. B. Golding, B. J. McConkey and D. M. E Bowdish, "The evolution of the class A scavenger receptors," *BMC evolutionary biology,* vol. 12, pp. 1--11, 2012.

[4] A. K. Wong, Z. Pei-Yuan and A. B. Zahid, "Pattern discovery and disentanglement on relational datasets," *Scientific Reports,* vol. 11, no. 1, p. 5688, 2021.

[5] W. Wolberg, "Breast Cancer Wisconsin (Original) Data Set," [Online]. Available: https://archive.ics.uci.edu/ml/datasets/breast+cancer+wisconsin+(original)..

[6] "Statlog (Heart) Data Set," [Online]. Available: https://archive.ics.uci.edu/ml/datasets/Statlog+(Heart)..

[7] A. Asuncion and D. Newman, "UCI Machine Learning Repository,," School of Information and Computer Science, University of California, Irvine, CA, 2007. [Online]. Available: http://archive.ics.uci.edu/ml/.

[8] "Thoracic Surgery Data Set," UCI Repository, November 2013. [Online]. Available: http://archive.ics.uci.edu/ml/datasets/Thoracic+Surgery+Data..
